# Characterization of *ZmPMP3g* function in drought tolerance of maize

**DOI:** 10.1038/s41598-023-32989-4

**Published:** 2023-05-05

**Authors:** Ling Lei, Hong Pan, Hai-Yang Hu, Xian-Wei Fan, Zhen-Bo Wu, You-Zhi Li

**Affiliations:** State Key Laboratory for Conservation and Utilization of Subtropical Agro-Bioresources/College of Life Science and Technology, Guangxi University, 100 Daxue Road, Nanning, 530004 Guangxi China

**Keywords:** Plant molecular biology, Plant stress responses

## Abstract

The genes enconding proteins containing plasma membrane proteolipid 3 (PMP3) domain are responsive to abiotic stresses, but their functions in maize drought tolerance remain largely unknown. In this study, the transgenic maize lines overexpressing maize *ZmPMP3g* gene were featured by enhanced drought tolerance; increases in total root length, activities of superoxide dismutase and catalase, and leaf water content; and decreases in leaf water potential, levels of O_2_^−·^and H_2_O_2_, and malondialdehyde content under drought. Under treatments with foliar spraying with abscisic acid (ABA), drought tolerance of both transgenic line Y7-1 overexpressing *ZmPMP3g* and wild type Ye478 was enhanced, of which Y7-1 showed an increased endogenous ABA and decreased endogenous gibberellin (GA) 1 (significantly) and GA3 (very slightly but not significantly) and Ye478 had a relatively lower ABA and no changes in GA1 and GA3. *ZmPMP3g* overexpression in Y7-1 affected the expression of multiple key transcription factor genes in ABA-dependent and -independent drought signaling pathways. These results indicate that *ZmPMP3g* overexpression plays a role in maize drought tolerance by harmonizing ABA-GA1-GA3 homeostasis/balance, improving root growth, enhancing antioxidant capacity, maintaining membrane lipid integrity, and regulating intracellular osmotic pressure. A working model on ABA-GA-Z*mPMP3g* was proposed and discussed.

## Introduction

The genes that encode proteins with a plasma membrane proteolipid 3 (PMP3) domain are present across prokaryotes and eukaryotes and frequently associated with responses to abiotic stresses, forming a PMP3 family^1,2^. The name of PMP3 proteins varies with species, such as low temperature inducible 6 (LTI6) and rare cold inducible 2 (RCI2) proteins due to their homology with AtRCI2A/B of Arabidopsis (*Arabidopsis thaliana*)^1,2^. PMP3/RCI2 proteins are small hydrophobic membrane proteins (about 52–64 amino acids in length) of two transmembrane domains and a hydrophilic C-terminal tail for almost half the RCI2s, which can be divided into differnt structural groups^1,2^. Because *PMP3*s/*RCI2*s are mostly found in response to salt stress they are often labelled as salt-tolerant genes in most cases although their expression is also markedly induced by cold and drought^1,2^.

*AtRCI2A* plays a role directly or indirectly for avoiding over-accumulation of excess Na^+^ and K^+^ ions at a cellular level, with contributions to salt tolerance at a whole plant level of Arabidopsis^1,2^. The enhanced salt tolerance of transgenic alfalfa chimera overexpressing alfalfa (*Medicago sativa* L.) genes of *MsRCI2D* and *MsRCI2E* is correlated with the increased enzyme activities of superoxide dismutase (SOD), peroxidase (POD), catalase (CAT), and glutathione reductase, and the lower Na^+^/K^+^ ratio in transgenic hairy roots^3^. Overexpressing *CsRCI2D* from *Camelina sativa* increases intracellular lipid content and therefore enhances high temperature tolerance of transgenic camelina lines^4^. The *AcPMP3-1* of halophyte *Aneurolepidium chinense* is essential for regulating Na^+^/K^+^ transportation between plant roots and the outer environment under salt stress^5^. Transgenic tobacco expressing *AlTMP1* of *Aeluropus littoralis* exhibits the enhanced tolerance to salt, drought, osmotic, H_2_O_2_, heat and freezing stresses at the seedling stage, however, which had a higher tolerance to drought than to salinity^6^.

In spite of their high similarity, conserved subcellular localization, and common origin, functional roles of different RCI2 proteins may be distinct^7^. Arabidopsis *AtRCI2*s except *AtRCI2C* and *AtRCI2H* of no functions are reported to contribute tolerance to cold, drought, and salt, and barley *Hvblt101.1* is found to function in cold and salt tolerance rather than drough tolerance^1^. Three alfalfa *MsRCI2*s, *MsRCI2A, MsRCI2B*, and *MsRCI2C*, have functional differences in tolerance to alkali and salt^8^. Anyway, the precise mechanisms that *RCI2*s contribute to abiotic stress tolerance remain largely unknown^2^.

Maize is one of the three major crops^9^. Identification of functional genes with drought tolerance is necessary and important for molecular breeding of maize drought tolerance because this crop is quite susceptible to drought^10^. So far, a total of 11 maize *PMP3*/*RCI2* genes are found^1^, including *ZmPMP3-1* to *ZmPMP3-8*, *ZmRCI2-3*, *ZmRCI2-8*, and *ZmRCI2-9*. Eight ZmPMP3 proteins are categorized into three groups^11^, of which ZmPMP3-1, ZmPMP3-5, ZmPMP3-7 and ZmPMP3-8 belong to the group I, ZmPMP3-4 and ZmPMP3-6 are in the group II, and ZmPMP3-2 is under the group III^11^. Up to now, *ZmPMP3*s are documented to function in salt tolerance^11^. The *ZmRCI2*s are considered to be related to drought tolerance because the expression is responsive to drought^12^. Nevertheless, there is no functional experimental evidence, and the detailed mechanisms of the *ZmPMP3*s in drought tolerance of maize are still unknown.

Based on the transcriptome of maize inbred line YQ7-96 under salt stress, we cloned an early drought stress-inducible gene (GenBank accession no. EC869579.1), numbered RA33G4 and now named *ZmPMP3g*, which encods a PMP3 domain protein^13^. We inferred that *ZmPMP3g* could likely confer drought tolerance of maize. To confirm the assumption, in the present study, *ZmPMP3g* function in drought tolerance of maize was investigated.

## Results

### *ZmPMP3g* gene

The *ZmPMP3g* had 99% sequence identity with a *LTI6B* (GenBank accession no. EU961419.1/EU954644.1) and 97% sequence identity with an early drought induced protein gene (GenBank accession no. NM_001114162) of maize at nucleotide level, respectively. The encoded protein ZmPMP3g was composed of 58 amino acid residues, showing 100% identity with an early drought-inducible protein (GenBank accession no. ABY71210.1) and ZmPMP3-1 protein (EU364508) of maize at amino acid level, respectively.

### Transgenic maize lines overexpressing *ZmPMP3g*

The *ZmPMP3g* gene was introduced into 4 Chinese maize elite inbred lines of Chang7-2 (C7-2), Huangzao4 (HZ4), Ye478 (Y48), and Zheng58 (Z58)^14^, respectively. The transformed plants were subjected to herbicide Basta resistance screening (Supplementary Fig. S1) and analyses of polymerase chain reaction (PCR) (Supplementary Fig. S2). A total of 7 transgenic lines were further identified by Southern blotting from T4-generation transgenic plants, which were numbered as C6-1, C3-1 and C-7-2 derived from C7-2, H2-3 derived from HZ4, Y7-1 derived from Y48, and Z1-3 and Z3-1 derived from Z58. Southern blotting indicated that there were 2 expression constructs inserted in C6-1 and Z1-3, and 1 expression construct inserted in C-7-2, C3-1, H2-3, Y7-1, and Z3-1 (Supplementary Fig. S3).

**Figure 1:**
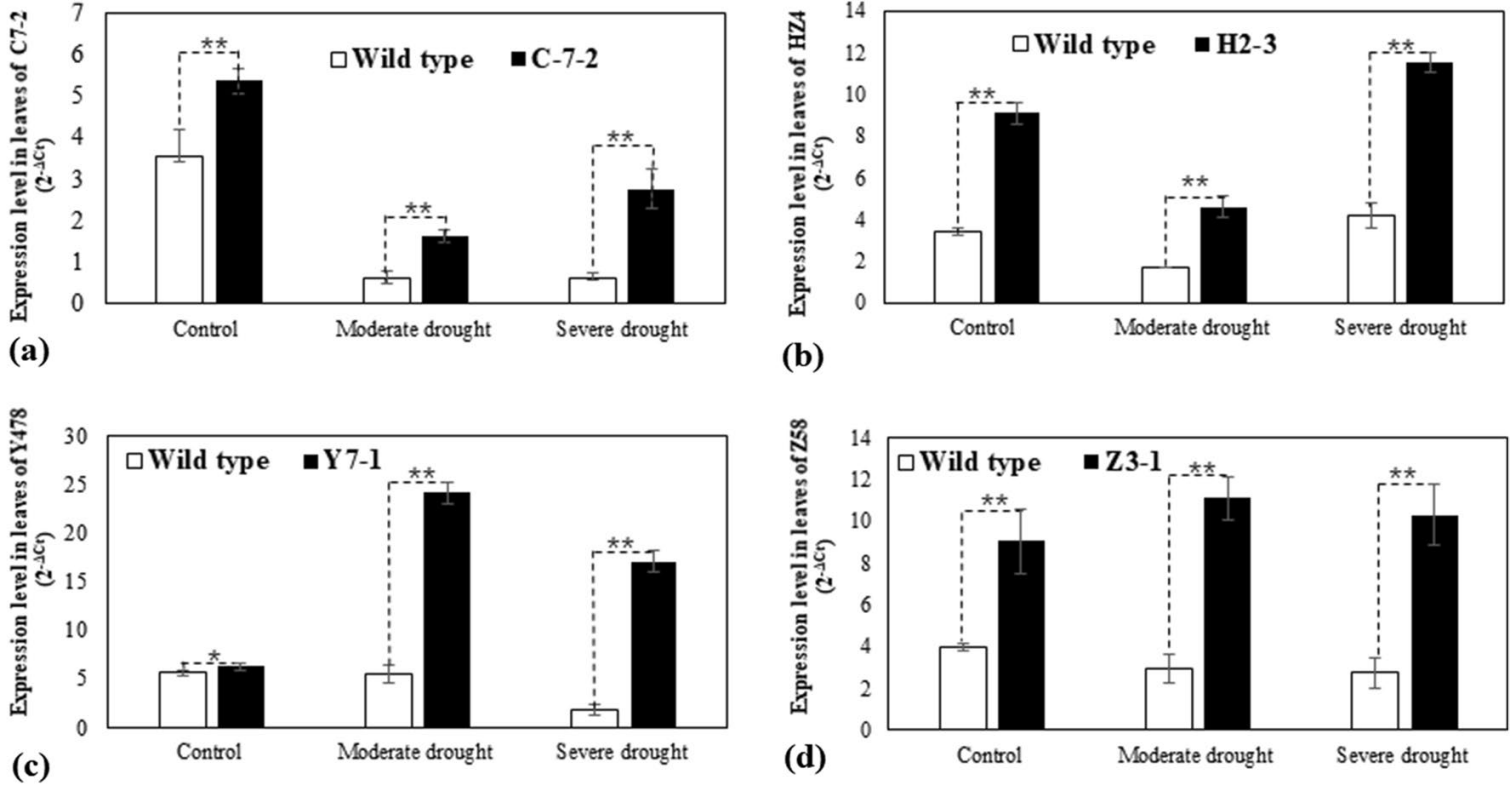
The expression of *ZmPMP3g* in pot-grown maize. The analyses were conducted by qPCR on the second fully-expanded leaves down from the top of the plants. Transgenic plants analyzed were from T4-generation lines. The leaf sampling was conducted on the second day after the pot mix moisture was at the upper threshold of 50% for moderate drought and 40% for severe drought, respectively. Each datum was the mean ± standard deviation (SD) from 3 individual plants selected at random. The single asterisk (*) and double asterisk (**) indicated significant differences at *p* < 0.05 and *p* < 0.01, respectively. C7-2, Maize inbred line Chang7-2; C-7-2, C7-2 overexpressing *ZmPMP3g*; HZ4, Maize inbred line Huangzao4; H2-3, HZ4 overexpressing *ZmPMP3g*; qPCR, Quantitative PCR; T4, Transgenic generation 4; Y478, Maize inbred line Ye478; Y7-1, Y478 overexpressing *ZmPMP3g*; Z58, Maize inbred line Z58; Z3-1, Z58 overexpressing *ZmPMP3g*.

The subsequent experiments focused on transgenic lines of C-7-2, H2-3, Y7-1, and Z3-1 containing 1 inserted expression construct. Quantitative PCR (qPCR) analysis showed that the *ZmPMP3g* expression level was significantly higher in T4-generation transgenic plants than in respective non-trangenic wild type plants (Fig. 1).

### Drought tolerance of maize

**Figure 2:**
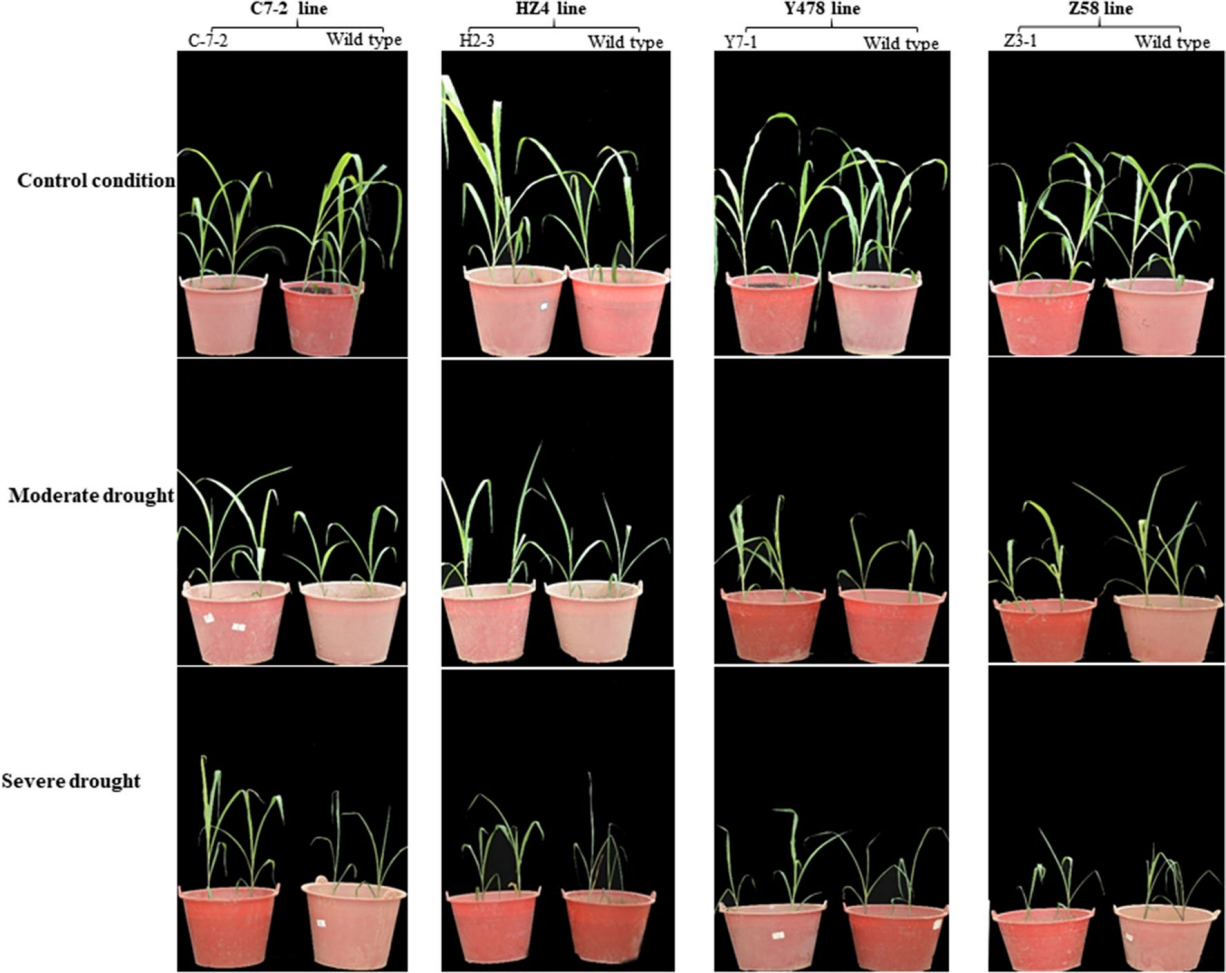
Drought tolerance phenotype of pot-grown maize plants. Photos were taken on the second day after the pot mix moisture was at the upper threshold of 50% for moderate drought and 40% for severe drought, respectively. Transgenic plants analyzed were from T4-generation lines. C7-2, Maize inbred line Chang7-2; C-7-2, C7-2 overexpressing *ZmPMP3g*; HZ4, Maize inbred line Huangzao4; H2-3, HZ4 overexpressing *ZmPMP3g*; T4, Transgenic generation 4; Y478, Maize inbred line Ye478; Y7-1, Y478 overexpressing *ZmPMP3g*; Z58, Maize inbred line Z58; Z3-1, Z58 overexpressing *ZmPMP3g*.

The transgenic plants were relatively more tolerant to drought than respective wild type plants especially under severe drought in pot experiments (Fig. 2).

**Figure 3:**
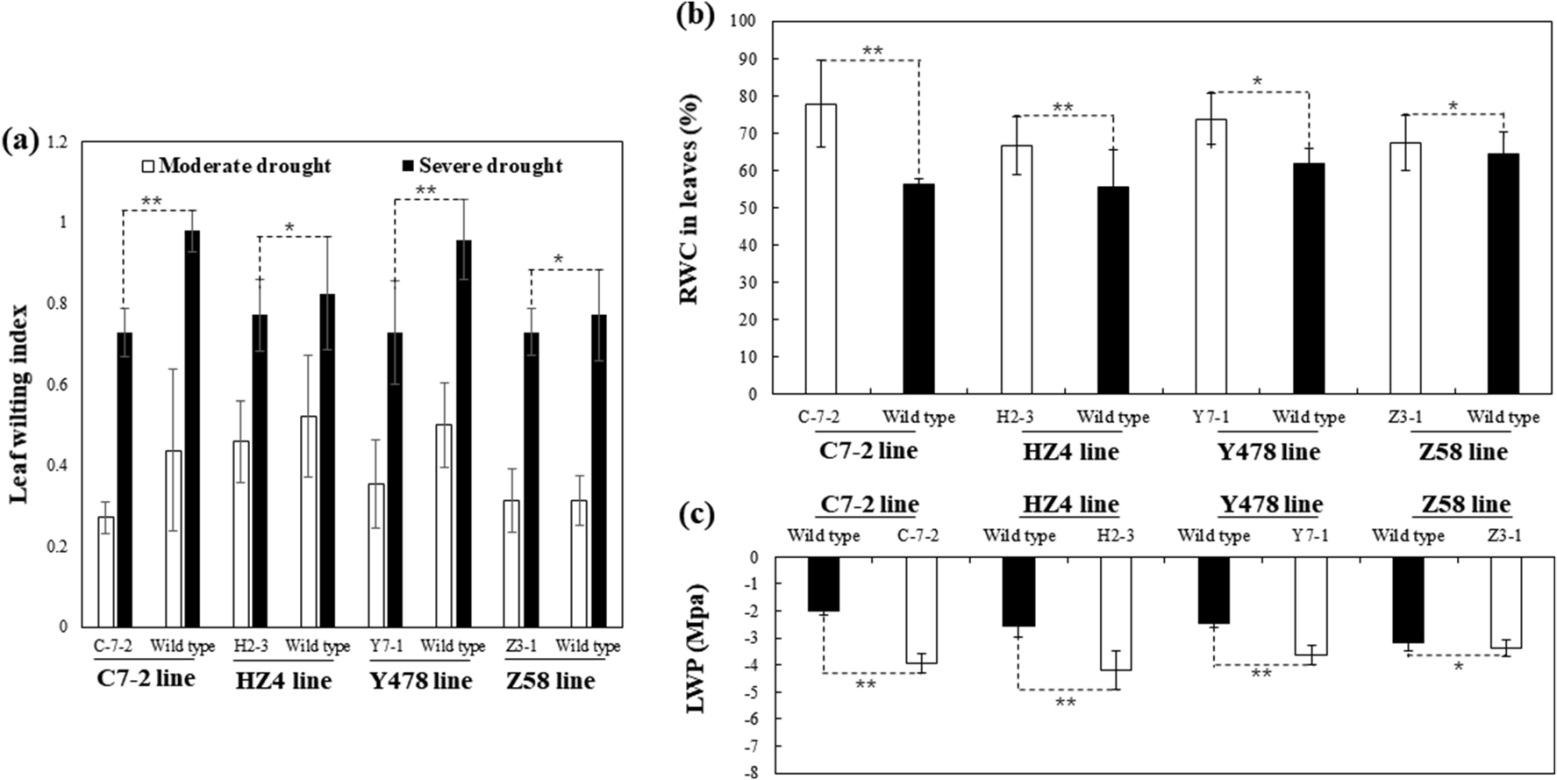
Leaf wilting index (**a**), leaf RWC (**b**), and LWP (**c**) of pot-grown maize. Analyses were conducted on the second day after the pot mix moisture was at the upper threshold of 50% for moderate drought and 40% for severe drought, respectively. Transgenic plants analyzed were from T4-generation lines. The leaf wilting index for each line under each treatment was calculated based on all leaves of 5 individual plants selected at random. The RWC and LWP were analzyed by using the second fully-expanded leaves down from the top of 3 individual plants selected at random under moderate drought and presented as the mean ± SD. The single asterisk (*) and double asterisk (**) indicated significant differences at *p* < 0.05 and *p* < 0.01, respectively. C7-2, Maize inbred line Chang7-2; C-7-2, C7-2 overexpressing *ZmPMP3g*; HZ4, Maize inbred line Huangzao4; H2-3, HZ4 overexpressing *ZmPMP3g*; LWP, Leaf water potential; RWC, Relative water content; SD, Standard deviation; T4, Transgenic generation 4; Y478, Maize inbred line Ye478; Y7-1, Y478 overexpressing *ZmPMP3g*; Z58, Maize inbred line Z58; Z3-1, Z58 overexpressing *ZmPMP3g*.

**Figure 4:**
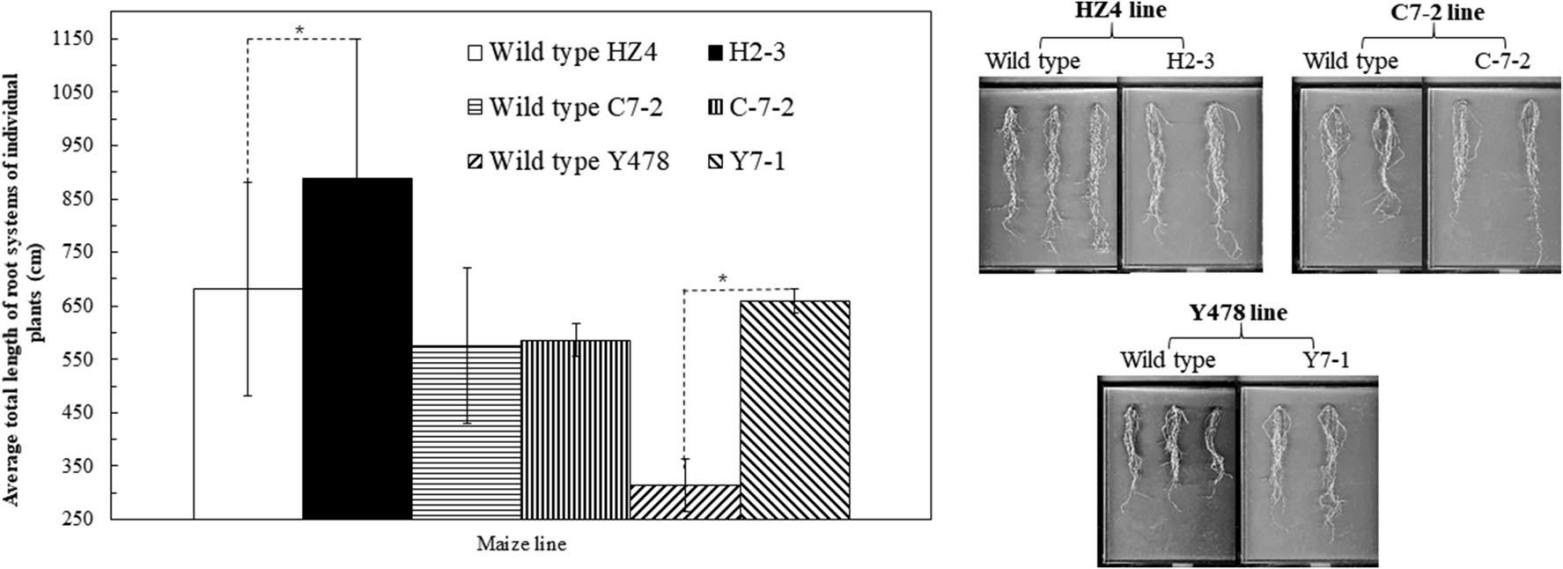
The total root length of pot-grown maize. Analyses were conducted on the third day after the pot mix moisture was at the upper threshold of 40% for severe drought. Transgenic plants analyzed were from T4-generation lines. The data of transgenic line Z3-1 and wild type Z58 were not available. Each datum was the mean ± SD from root systems of 5 individual plants selected at random. The single asterisk (*) indicated a significant difference at *p* < 0.05. C7-2, Maize inbred line Chang7-2; C-7-2, C7-2 overexpressing *ZmPMP3g*; HZ4, Maize inbred line Huangzao4; H2-3, HZ4 overexpressing *ZmPMP3g*; SD, Standard deviation; T4, Transgenic generation 4; Y478, Maize inbred line Ye478; Y7-1, Y478 overexpressing *ZmPMP3g*; Z58, Maize inbred line Zheng58; Z3-1, Z58 overexpressing *ZmPMP3g*.

The transgenic plants had a lower leaf wilting index (Fig. 3a), higher leaf relative water contents (RWC) (Fig. 3b), and lower leaf water potentials (LWP) under drought (Fig. 3c). The average total root length was found to significantly increase in transgenic lines of H2-3 and Y7-1, and slightly but not significantly in transgenic line C-7-2 under drought (Fig. 4).

### Betaine and total sugar

**Figure 5:**
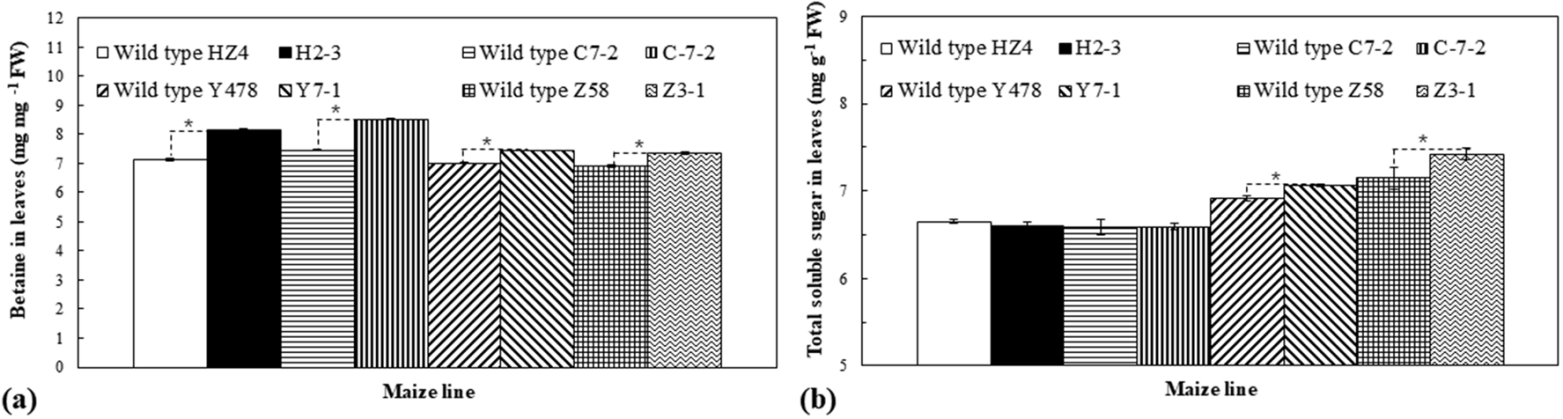
The contents of betaine (**a**) and total soluble sugar (**b**) of pot-grown maize. Analyses were conducted on the third day after the pot mix moisture was at the upper threshold of 40% for severe drought. Transgenic plants analyzed were from T4-generation lines. Each datum was the mean ± SD from root systems of 3 individual plants selected at random. The single asterisk (*) indicated a significant difference at *p* < 0.05. C7-2, Maize inbred line Chang7-2; C-7-2, C7-2 overexpressing *ZmPMP3g*; FW, Fresh weight; HZ4, Maize inbred line Huangzao4; H2-3, HZ4 overexpressing *ZmPMP3g*; SD, Standard deviation; T4, Transgenic generation 4; Y478, Maize inbred line Ye478; Y7-1, Y478 overexpressing *ZmPMP3g*; Z58, Maize inbred line Z58; Z3-1, Z58 overexpressing *ZmPMP3g*.

The betaine content significantly increased in leaves of the transgenic plants (Fig. 5a). The total soluble sugar content had no significant changes in leaves of transgenic lines of H2-3 and C-7-2 but significantly increased in leaves of transgenic lines of Y7-1 and Z3-1 (Fig. 5b).

### Reactive oxygen species (ROS), malondialdehyde (MDA), and antioxidant enzyme activity

**Figure 6:**
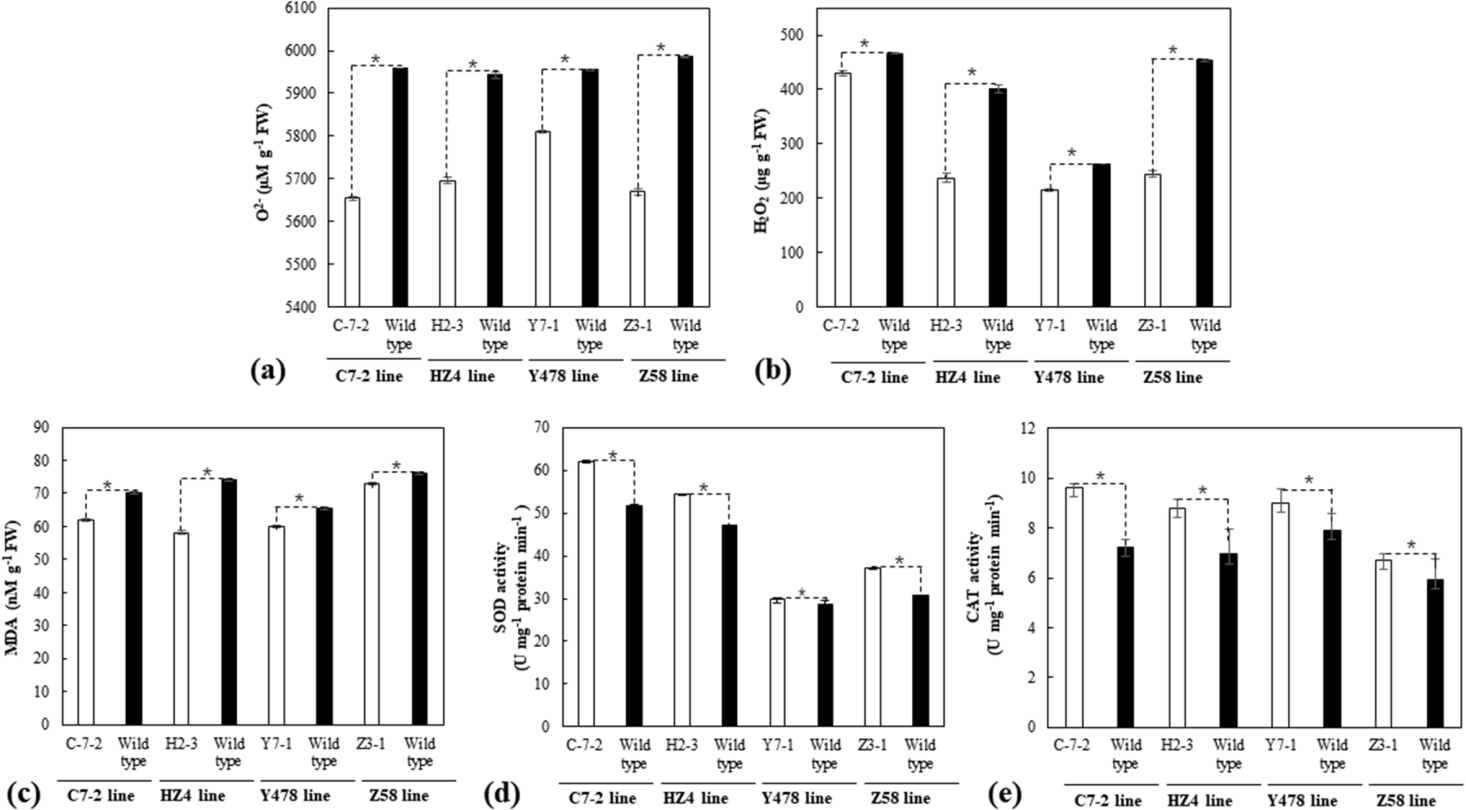
The contents of O_2_^−·^ (**a**), H_2_O_2_ (**b**), MDA (**c**), SOD (**d**), and CAT (**e**) in pot-grown maize. Analyses were conducted on the second day after the pot mix moisture was at the upper threshold of 40% for severe drought. Transgenic plants analyzed were from T4-generation lines. Each datum was the mean ± SD from the second fully-expanded leaves down from the top of 3 individual plants selected at random. The single asterisk (*) indicated a significant difference at *p* < 0.05. CAT, Catalase; C7-2, Maize inbred line Chang7-2; C-7-2, C7-2 overexpressing *ZmPMP3g*; FW, Fresh weight; HZ4, Maize inbred line Huangzao4; H2-3, HZ4 overexpressing *ZmPMP3g*; MDA, Malondialdehyde; SD, Standard deviation; SOD, Superoxide dismutase; T4, Transgenic generation 4; Y478, Maize inbred line Ye478; Y7-1, Y478 overexpressing *ZmPMP3g*; Z58, Maize inbred line Z58; Z3-1, Z58 overexpressing *ZmPMP3g*.

The leaves of the transgenic plants showed lower contents of O_2_^−·^ (Fig. 6a), H_2_O_2_ (Fig. 6b) and MDA (Fig. 6c), and higher activities of antioxidant enzymes SOD (Fig. 6d) and CAT (Fig. 6e) than those in leaves of respective wild type lines when pot-grown under severe drought.

### Growth of Y7-1 and Y478 under exogenous ABA

**Figure 7:**
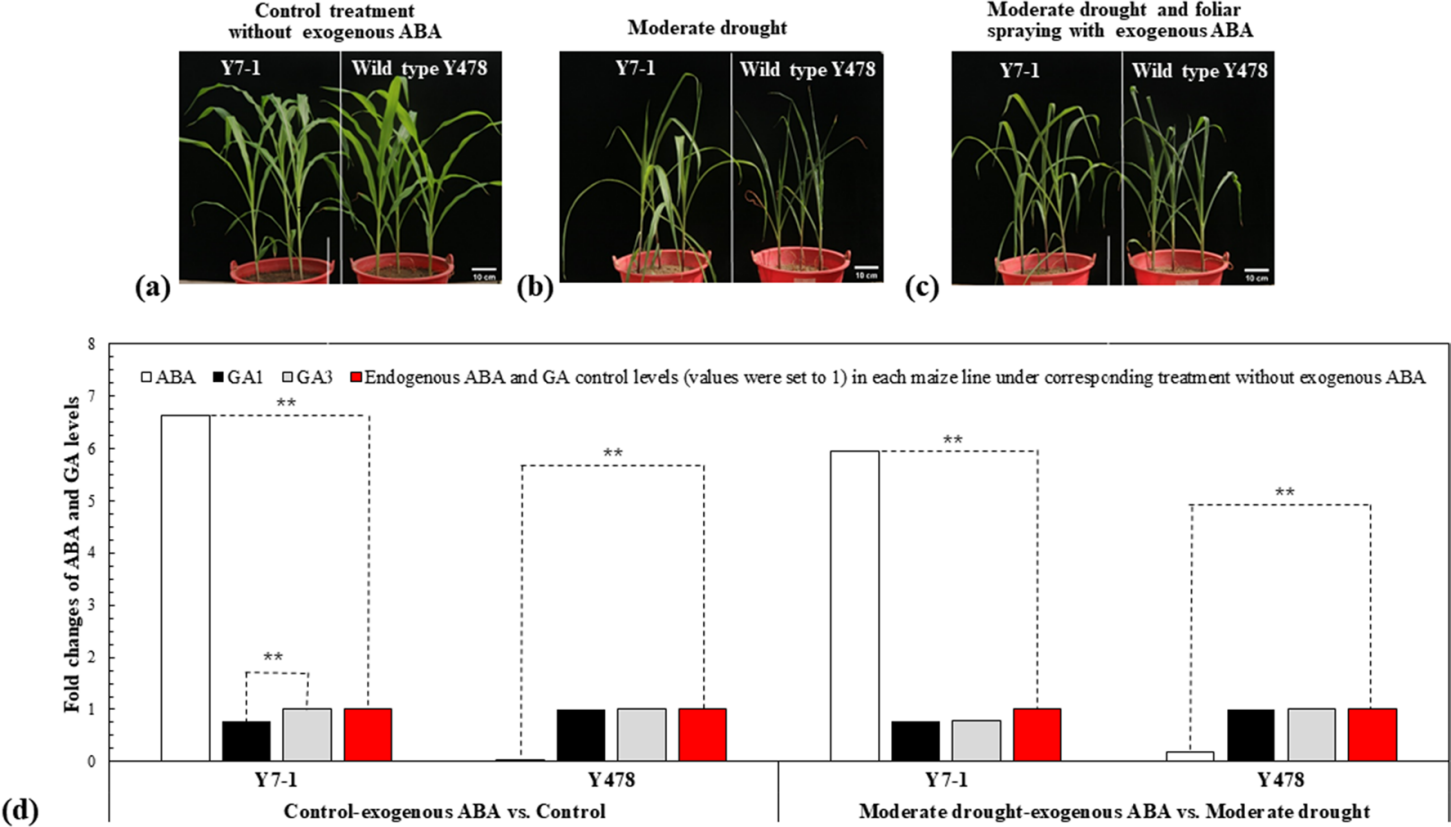
Phenotypes (**a**–**c**), and GA1 and GA3 level changes (**d**) of transgenic line Y7-1 and wild type Y478 pot-grown under treatments without or with foliar spraying with exogenous ABA. In (**a**–**c**), analyses were conducted after 9 days of foliar spraying with exogenous ABA. Y7-1 was T4-generation lines. Spraying with exogenous ABA started on the first day after the pot mix moisture was at the upper thresholds of 50%. In (**d**), the second fully-expanded leaves down from the tope of 3 individual plants selected at random were analyzed, where each datum represented the the fold value of change under foliar spraying with exogenous ABA in comparison with that in respective maize line under treatments without exogenous ABA. The red columns were used as the control levels of endogenous GA and ABA, where the values under corresponding treatments without exogenous ABA were set to 1. The double asterisk (**) indicated a significant difference at *p* < 0.01. ABA, Abscisic acid; GA, Gibberellin; Y478, Maize inbred line Ye478; Y7-1, Y478 overexpressing *ZmPMP3g*.

The transgenic line Y7-1 and the corresponding wild type Y478 grew better under control treatment (Fig. 7a) than under moderate drought treatments without (Fig. 7b) or with spraying with exogenous ABA (Fig. 7c). Either Y7-1 or Y478 grew better under a combined treatment of moderate drought and exogenous ABA (Fig. 7c) than under moderate drought treatment (Fig. 7b). Anyway, Y7-1 was more tolerant to drought than Y478 (Fig. 7b,c).

### The expression of genes related to ABA-dependent and -independent signaling (ADIDS) pathways in Y7-1 and Y478

Transcriptome sequencing analyses were conducted on leaves of transgenic line Y7-1 and wild type Y478 pot-grown under control, both control and exogenous ABA (control-exogenous ABA), moderate drought, and moderate drought-exogenous ABA, respectively. As a result, application of exogenous ABA did not lead to significant changes in the expression of a *ZmPMP3g* homolog (Zm00001d024778) in Y7-1 and Y478 under treatments with exogenous ABA against treatments without exogenous ABA.

There were 74 differentially expressed genes (DEG) related to ADIDS found in Y478 leaves under moderate drought against control (Supplementary Table S1), 288 ADIDS-related DEGs in Y478 leaves under moderate drought-exogenous ABA against moderate drought (Supplementary Table S2), and 4 ADIDS-related DEGs in Y478 leaves under control-ABA against control (Supplementary Table S3). There were 59 ADIDS-related DEGs found in Y7-1 leaves against Y478 under control (Table 1). However, the number of the related DEGs was much lower in Y7-1 under treatments with exogenous ABA than under treatments without exogenous ABA (Table 2). Of the DEGs, many belonged to transcription factor genes of *AP2-EREBP*, *bHLH*, *WRKY*, *bZIP*, *NAC*, *SNF*, *SnRK*, *VIVIPAROUS1*, *RPE*, *DREB*, and *ERF* families.

**Table 1:** The DEGs related to ADIDS in leaves of T4-generation transgenic line Y7-1 under control alone as control (Y7-1) vs. control (Y478) in pot experiments without foliar spraying with exogenous ABA.

Gene ID	Gene description	log2 (fold change)^a^	padj
Zm00001d015779	14-3-3-like protein GF14 omega	7.18	0.001
Zm00001d052338	AP-1 complex subunit mu-2	6.65	0.000
Zm00001d042640	SnRK1-interacting protein 1	6.23	0.003
Zm00001d033471	Transcription factor bHLH112	5.52	0.027
Zm00001d044857	AP2-EREBP-transcription factor 31	4.58	0.000
Zm00001d028664	Transcription factor bHLH112	4.22	0.000
Zm00001d030995	bZIP-transcription factor 111	3.43	0.000
Zm00001d021164	bHLH-transcription factor 109	3.08	0.041
Zm00001d012237	SnRK1-interacting protein 1	2.26	0.000
Zm00001d024784	MYB-transcription factor 149	2.22	0.013
Zm00001d017766	nine-cis-epoxycarotenoid dioxygenase8	1.94	0.000
Zm00001d002143	bZIP-transcription factor 27	1.91	0.000
Zm00001d010445	Abscisic acid receptor PYL9	1.88	0.000
Zm00001d028962	WRKY-transcription factor 32	1.86	0.000
Zm00001d050893	NAC-transcription factor 40	1.81	0.000
Zm00001d003712	abscisic acid stress ripening3	1.59	0.002
Zm00001d035512	AP2-EREBP-transcription factor 81	1.58	0.000
Zm00001d053988	bZIP-transcription factor 84	1.55	0.000
Zm00001d032024	myb transcription factor38	1.50	0.001
Zm00001d023529	abscisic acid stress ripening1	1.23	0.004
Zm00001d013130	bHLH-transcription factor 60	1.20	0.022
Zm00001d042695	SnRK2 serine threonine protein kinase 4	1.13	0.002
Zm00001d012067	bHLH-transcription factor 55	1.13	0.007
Zm00001d051554	abscisic acid 8'-hydroxylase2	1.13	0.030
Zm00001d021019	bHLH-transcription factor 136	1.04	0.010
Zm00001d015407	G2-like-transcription factor 53	− 1.05	0.001
Zm00001d028297	bHLH-transcription factor 3	− 1.09	0.005
Zm00001d043153	bZIP-transcription factor 56	− 1.17	0.023
Zm00001d011969	NAC-transcription factor 9	− 1.18	0.000
Zm00001d022542	bZIP-transcription factor 54	− 1.20	0.000
Zm00001d053124	MYB-transcription factor 61	− 1.22	0.001
Zm00001d038357	bHLH-transcription factor 118	− 1.23	0.000
Zm00001d038585	AP2-EREBP-transcription factor 196	− 1.29	0.011
Zm00001d008968	AP2-EREBP-transcription factor 96	− 1.32	0.033
Zm00001d018178	bZIP-transcription factor 4	− 1.33	0.000
Zm00001d040318	NAC-transcription factor 66	− 1.38	0.000
Zm00001d034447	bZIP-transcription factor 41	− 1.44	0.042
Zm00001d018081	AP2-EREBP-transcription factor 18	− 1.50	0.001
Zm00001d044065	DELLA protein RGA	− 1.54	0.047
Zm00001d009088	myb transcription factor95	− 1.59	0.037
Zm00001d032694	MYB-transcription factor 121	− 1.60	0.000
Zm00001d053707	AP2-EREBP-transcription factor 8	− 1.63	0.000
Zm00001d007962	G2-like-transcription factor 27	− 1.64	0.000
Zm00001d012585	AP2-EREBP-transcription factor 83	− 1.69	0.001
Zm00001d036736	bZIP-transcription factor 76	− 2.02	0.000
Zm00001d038221	NAC-transcription factor 20	− 2.18	0.000
Zm00001d044242	bHLH-transcription factor 25	− 2.35	0.000
Zm00001d016873	bHLH-transcription factor 152	− 3.36	0.000
Zm00001d025910	AP2-EREBP-transcription factor 86	− 3.65	0.000
Zm00001d044409	MYB-related-transcription factor 17	− 3.96	0.000
Zm00001d036768	MYB-transcription factor 158	− 4.28	0.000
Zm00001d028178	NAC-transcription factor 64	− 4.51	0.000
Zm00001d009468	AP2-EREBP-transcription factor 49	− 4.74	0.000
Zm00001d037531	AP2-EREBP-transcription factor 9	− 5.46	0.006
Zm00001d009622	AP2-EREBP-transcription factor 12	− 7.99	0.000
Zm00001d003512	Zeaxanthin epoxidase chloroplastic	− 1.04	0.006
Zm00001d014701	Myb family transcription factor PHL6	− 1.20	0.001
Zm00001d007446	14-3-3-like protein	− 1.46	0.000
Zm00001d016105	Abscisic acid receptor PYL9	− 1.50	0.000
Zm00001d009090	Transcription factor MYB12	− 6.93	0.000

The results were based on transcriptome sequencing of the second fully-expanded leaves down from the top of 3 individual plants 9 d after the pot mix moisture was at the upper threshold of 50% for moderate drought.

ABA, Abscisic acid; ADIDS, ABA-dependent and independent signaling; DEG, Differentially expressed gene; padj: Adjust *p*-value; T4, Transgenic generation 4; Y478, Maize inbred line Ye478; Y7-1, Y478 overexpressing *ZmPMP3g.*

^a^Positive and negative values indicated up-regulation and down-regulation of gene expression, respectively.

**Table 2:** The DEGs related to ADIDS in leaves of T4-generation transgenic line Y7-1 in pot experiments with foliar spraying with exogenous ABA.

Gene ID	Gene description	log2 (fold change)^a^	padj
Under moderate drought as drought (Y7-1) vs. control (Y7-1)			
Zm00001d045463	NAC-transcription factor 86	3.42	0.004
Zm00001d000291	bHLH-transcription factor 88	2.70	0.000
Zm00001d033222	viviparous14	1.64	0.010
Zm00001d041472	NAC-transcription factor 108	1.44	0.029
Zm00001d042695	SnRK2 serine threonine protein kinase 4	1.21	0.025
Zm00001d007446	14-3-3-like protein	1.11	0.019
Zm00001d003401	General regulatory factor1	− 1.13	0.006
Zm00001d040621	myb transcription factor40	− 1.33	0.029
Zm00001d012527	NAC-transcription factor 23	− 1.42	0.037
Zm00001d038207	NAC-transcription factor 3	− 1.43	0.013
Zm00001d023411	G2-like-transcription factor 5	− 1.55	0.048
Zm00001d021019	bHLH-transcription factor 136	− 1.57	0.001
Zm00001d006065	bHLH-transcription factor 121	− 1.89	0.029
Zm00001d019207	NAC-transcription factor 4	− 2.04	0.010
Zm00001d021164	bHLH-transcription factor 109	− 4.68	0.001
Zm00001d032144	AP2-EREBP-transcription factor 60	− 4.75	0.000
Under moderate drought-exogenous ABA as moderate drough-exogenous ABA (Y7-1) vs. drought (Y7-1)			
Zm00001d004843	Ascisic acid stress ripening2	1.92	0.036
Zm00001d003712	Abscisic acid stress ripening3	1.51	0.000
Zm00001d041576	MYB-transcription factor 6	− 1.06	0.046
Zm00001d040536	bHLH-transcription factor 68	− 1.46	0.012
Zm00001d026398	bZIP-transcription factor 113	− 1.52	0.001
Zm00001d030995	bZIP-transcription factor 111	− 1.66	0.000
Zm00001d036551	MYB-transcription factor 59	− 3.73	0.033
Zm00001d025401	Abscisic acid stress ripening5	− 4.58	0.026
Under control-exogenous ABA as control-exogenous ABA (Y7-1) vs. control (Y7-1)			
Zm00001d003712	Abscisic acid stress ripening3	− 1.56	0.02

Foliar spraying with ABA started on the first day when the pot mix moisture was at the upper threshold of 50% for moderate drought. The results were based on transcriptome sequencing of the second fully-expanded leaves down from the top of 3 individual plants 9 d after the pot mix moisture was at the upper threshold of 50% for moderate drought.

ABA, Abscisic acid; ADIDS, ABA-dependent and -independent signaling; DEG, Differentially expressed gene; padj: Adjust *p*-value; T4, Transgenic generation 4; Y478, Maize inbred line Ye478; Y7-1, Y478 overexpressing *ZmPMP3g.*

^a^Positive and negative values indicated up-regulation and down-regulation of gene expression, respectively.

### The expression of genes related to gibberellin (GA) in Y7-1

By results of transcriptome sequencing, DEGs related to GA biosynthesis and catabolism were found (Table 3). Of these genes, the expression of *GA2ox7*, *GA2ox12* and *GA20ox5* was up-regulated. The expression of one DELLA protein RGA gene was found to be down-regulated in Y7-1 against Y478 under the moderate drought-exogenous ABA treatment (Table 3). Several DEGs encoding Skp1, CUL1, and F-box proteins, which were related to DELLA degradation, were found in Y7-1 (Table 3).

**Table 3:** The DEGs related to GA biosynthesis in leaves of T4-generation transgenic line Y7-1 in pot experiments with foliar spraying with exogenous ABA.

Gene ID	Gene description	log2 (fold change)^a^	padj
Under moderate drought-exogenous ABA as moderate drought-exogenous ABA (Y7-1) vs. moderate drought-exogenous ABA (Y478)			
Zm00001d018617	GA2ox 12	5.19	0.000
Zm00001d038695	GA2ox 7	3.49	0.003
Zm00001d012212	GA20ox 5	1.33	0.018
Zm00001d050493	Gibberellin receptor GID1L2	1.22	0.003
Zm00001d016895	Gibberellin receptor GID1L2	3.24	0.000
Zm00001d044065	DELLA protein RGA	− 2.19	0.044
Zm00001d016052	WRKY transcription factor 22	− 1.38	0.028
Zm00001d039245	WRKY transcription factor 6	− 1.80	0.000
Zm00001d028962	WRKY55	− 2.42	0.000
Zm00001d013738	Fcf2 pre-rRNA processing protein	9.78	0.000
Zm00001d045882	F-box family protein	6.27	0.001
Zm00001d015791	F-box domain protein	6.12	0.002
Zm00001d053172	F-box protein PP2-B10	3.82	0.000
Zm00001d016066	F-box/kelch-repeat protein	1.69	0.000
Zm00001d000224	F-box protein SKP2A	1.23	0.000
Zm00001d016553	F-box/kelch-repeat protein	1.15	0.000
Zm00001d029673	F-box protein PP2-A13	− 1.33	0.000
Zm00001d018452	F-box protein PP2-B10	− 1.46	0.001
Zm00001d033012	F-box/kelch-repeat protein SKIP11	-1.55	0.000
Zm00001d035303	F-box protein	− 1.64	0.000
Zm00001d028957	F-box protein	− 1.69	0.043
Zm00001d043989	F-box/kelch-repeat protein	− 1.85	0.016
Zm00001d047573	F-box protein	− 2.10	0.000
Zm00001d035636	F-box protein PP2-A13	− 6.18	0.001
Under moderate drought-exogenous ABA as moderate drought-exogenous ABA (Y7-1) vs. moderate drought (Y7-1)			
Zm00001d044680	WRKY-transcription factor 5	7.37	0.001
Zm00001d019542	COP1	1.12	0.000
Zm00001d025407	COP9 signalosome complex subunit 8	− 9.97	0.000
Zm00001d027974	Cullin-associated NEDD8-dissociated protein 1	5.11	0.032
Under control-exogenous ABA as control-exogenous ABA (Y7-1) vs. control (Y7-1)			
Zm00001d028962	WRKY-transcription factor 32	− 2.57	0.00
Zm00001d028957	F-box protein	− 2.37	0.023
Zm00001d047573	F-box protein	− 1.85	0.039

Foliar spraying with ABA started on the first day when the pot mix moisture was at the upper threshold of 50% for moderate drought. The results were based on transcriptome sequencing of the second fully-expanded leaves down from the top of 3 individual plants 9 d after the pot mix moisture was at the upper threshold of 50% for moderate drought.

ABA, Abscisic acid; DEG, Differentially expressed gene; GA, Gibberellin; GA2ox, GA2-oxidase; GA20ox, GA 20-oxidase; padj: Adjust *p*-value; T4, Transgenic generation 4; Y478, Maize inbred line Ye478; Y7-1, Y478 overexpressing *ZmPMP3g.*

^a^Positive and negative values indicated up-regulation and down-regulation of gene expression, respectively.

### Changes in endogenous GA and ABA levels in Y7-1 and Y478

GA and ABA contents were also analyzed with leaves of transgenic line Y7-1 and wild type Y478 pot-grown under control, control-exogenous ABA, moderate drought, and moderate drought-exogenous ABA, respectively. In order to reflect the change tendency of GA and ABA, the fold changes in ABA and GA levels under treatments with foliar spraying with exogenous ABA in comparison with those in respective maize line under treatments without exogenous ABA were analyzed if the endogenous ABA and GA levels under corresponding treatments without exogenous ABA were set to 1 as control. As a result, the endogenous ABA levels significantly increased, GA1 levels decreased significantly under control-exogenous ABA treatment and slightly but not significantly under moderate drought-exogenous ABA treatment, and GA3 levels decreased very slightly but not significantly, in leaves of Y7-1 (Fig. 7d). However, endogenous ABA levels were relatively lower, and both GA1 and GA3 levels did not significantly change in leaves of Y478 under treatments (Fig. 7d).

## Discussion

*ZmPMP3g* was a homologous gene of *ZmPMP3-1*, which belongs to the group I in *ZmPMP3* family according to amino acid sequence^11^. The enhanced drought tolerance (Fig. 2) and the increased total root length (Fig. 4) of transgenic maize lines suggest that *ZmPMP3g* plays a role in both drought tolerance and root growth of maize. The increase in root length is undoubtedly beneficial to the uptake and utilization of water in deep soil by plants under drought, which is usually driven by hydrotropism^15^. Taken together with the function of maize *ZmPMP3-1* in salt tolerance^11^, it can be concluded that *ZmPMP3* genes in the group I have multiple functional features.

Increases in leaf RWCs (Fig. 3b) and contents of betaine and total soluble sugars (Fig. 5) as abiotic stress tolerance-related osmoprotectants^16^ along with the decreased leaf wilting index (Fig. 3a) and LWP (Fig. 3c) in transgenic maize lines strongly evidence that *ZmPMP3g* expression can maintain the cell osmotic potential and water potential under drought. However, on the whole, these parameters’ changes were not absolutely proportional to the expression level of *ZmPMP3g* (Fig. 1) and even drought tolerance phenotypes (Fig. 2), implying that the *ZmPMP3g* expression would have the functionally different effects among maize lines differing in genetic backgrounds.

Abiotic stresses usually induce the overproduction of ROS such as O_2_^−·^ and H_2_O_2_, which would cause membrane lipid degradatation and then result in production of MDA^17–19^. O_2_^−·^ and H_2_O_2_, can be degraded by SOD and CAT^20^, respectively. The correlation of the decresed contents of O_2_^−·^ (Fig. 6a), H_2_O_2_ (Fig. 6b), and MDA (Fig. 6c) with the increased activites of SOD (Fig. 6d) and CAT (Fig. 6e) in all transgenic maize lines under drought suggest that *ZmPMP3g* can enhance antioxidant capacity and maintain membrane lipid integrity of maize under drought.

Plant tolerance to abiotic stresses is closely related to ADIDS pathways^21^, involving multiple transcription factor genes such as *AP2-EREBP*, *bHLH*, *MYB*s, *WRKY*s, *bZIP*s, NACs, *SNF*s, *SnRK*s, *RPE*s, *DREB*s, and *ERF*s^21–33^. Of them, *AP2/ERF*s, *bHLH*s, *DREB2*, and *NAC*s are the downstream genes of ABA-independent signaling pathway, and *MYB*s, *bZIP*s and *MYC*s are the downstream genes of ABA-dependent signaling pathway^28^. Nine-*cis*-epoxycarotenoid dioxygenases (NCED)/viviparous are enzymes responsible for ABA biosynthesis^29,34^ and related to ABA–independent signaling pathway by activating NAC proteins^29^. Our results strongly indicate that *ZmPMP3g* overexpression in transgenic line Y7-1 affects ADIDS pathways by influencing the expression of NCED8, PYL9 (a ABA receptor), viviparous, NAC and AP2/DREB genes (Tables 1 and 2), very similar to actions of Arabidoposis *AtRCI2A/B*^1^ and maize *ZmPMP3*-*1*^11^.

It has been found there is a autoregulatory negative feedback path between *MYC2* and phytohormone jasmonate to terminate the jasmonate signaling in tomato^35^. The number of DEGs involved in ADIDS pathways obviously tended to decrease in both transgenic Y7-1 (Table 2) and wildtype Y478 (Supplementary Table S3) under exogenous ABA, indicating that the enhanced ABA levels probably repress expression of the related genes in ADIDS pathways, in turn, the expression of some genes affected by* ZmPMP3g* overexpression would trigger ABA production. It could be therefore speculated that there exists a autoregulatory feedback path associated with *ZmPMP3g* overexpression, from ABA production to the gene expression in ADIDS pathways.

GAs can relieve from growth restraint by acting degradation of the growth repressor DELLA^36^, of which GA1 and GA3 are major hormones in the cytosol for plant growth^37^. DELLA degradation is also involved in COP1, FKF1/F-box, skp1, CUL1, and F-box proteins^38^. However, accumulating evidence shows that GA and ABA exert an antagonistic effect and need a balance/homeostasis in plants^39–42^, high GA and low ABA levels under favorable conditions and the reverse ratio under unfavorable conditions^43–45^. This is roughly consistent with our findings in this study (Fig. 7d), indicating that *ZmPMP3g* overexpression can harmonize the ABA-GA1-GA3 (especially GA1) balance, somewhat different from the homeostasis of ABA-GA3 in melon (*Cucumis melo*) seeds under treatments with exogenous ABA^45^ and ABA-GA3-GA4 (especially GA4) during early growth stage of cucumber (*C. sativus*) treated by melatonin under salt^43^.

**Figure 8:**
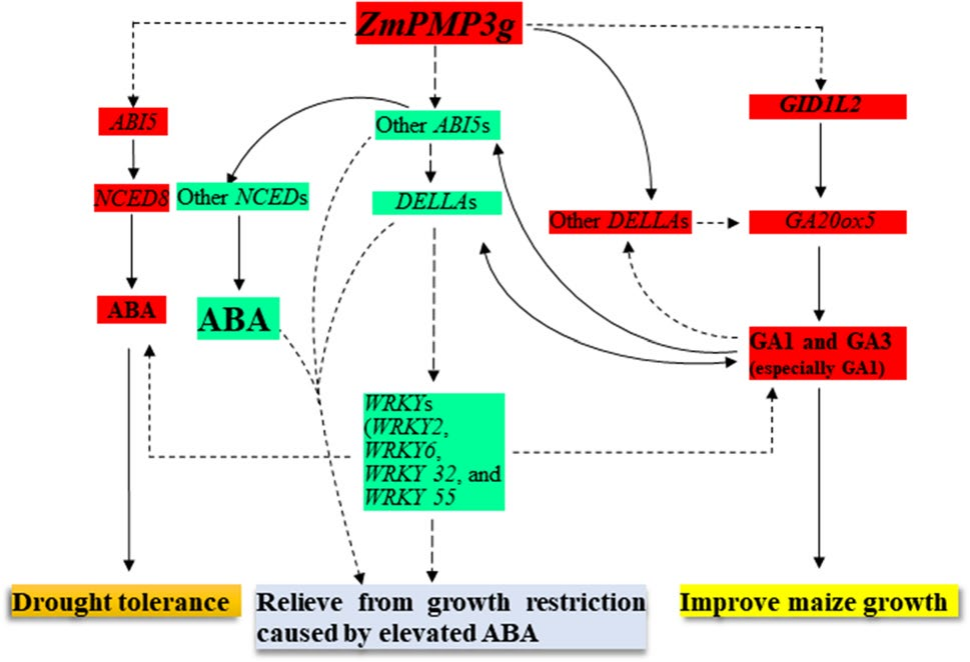
A working model related to *ZmPMP3g* overexpression. This model was established based on gene expression data in this study, of which the effects and paths of gene and enzyme actions referred to the literature^36–38,46,47,49,50,61–65^. Enhanced expression of *ZmPMP3g* gene would result in three major effects toward the following routes: (1) repressing the expression of some *ABI5*s, *DELLA*s, and *WRKY*s (*WRKY2*, *WRKY6*, *WRKY32* and *WRKY55*), and therefore relieving from maize growth restriction caused by elevated ABA; (2) inducing expression of *ABI5* and *NCED8*, promoting ABA production, and consequently endowing maize with drought tolerance; and (3) facilitating expression of *GID12* and *GA20ox5,* and GA1 production, and therefore improving maize growth under drought. In these processes, the cross-talk between the expression of related genes and production of ABA and GA occurred. The red and green boxes indicated an up-regulation/increase and a down-regulation/decrease in gene expression/ABA or GA production, respectively. The dashed lines with arrows denoted that the routes and/or their actions are unknown. The italics indicated genes. ABA, Abscisic acid; *ABI5,* ABA-insensitive 5; GA, Gibberellin; GA2ox, GA2-oxidase; GA20ox, GA20-oxidase; GID1L2, GA receptor; NCED*,* Nine-*cis*-epoxycarotenoid dioxygenase. *WRKY*, WRKY transcription factor.

In addition, many key regulator and/or receptor genes have been found involving GA-ABA homeostasis, including *DELLA*, *WRKY*, *ABA-insensitive* (*ABI*)*4* (*ABI4*), *ABI5*, and GA receptor *GID1L2*^44,46–50^. Rice *OsWRKY24* expression inhibits both GA and ABA signaling^46^. *ABI4* expression can enhance the expression of both *NCED6* related to ABA biosynthesis and *GA2ox7* controlling GA biosynthesis rate-limiting step^47^. Overexpression^48^ and repression^49^ of *ABI5* lead to a hypersensitiy and less sensitivity of Arabidopsis to exogenous ABA, respectively. Rice *GID1L2* mutant failes to respond to GA signaling^50^. Taken these reults together with our transcriptome data (Tables 1, 2, 3, 4; Supplementary Tables S1–S3), and changes in ABA and GA levels (Fig. 7d), a working model that the *ZmPMP3g* overexpression affects growth and drought tolerance of maize was proposed (Fig. 8).

**Table 4:** The DEGs encoding ABI proteins in GA biosynthesis in leaves of T4-generation transgenic line Y7-1 and wild type Y478 in pot experiments with foliar spraying with exogenous ABA.

Gene ID	Gene description	log2 (fold change)^a^	padj
In Y7-1 under moderate drought-exogenous ABA as moderate drought-exogenous ABA (Y7-1) vs. moderate drought-exogenous ABA (Y478)			
Zm00001d018178	ABI 5-like protein 5	− 1.76	0.000
Zm00001d020711	ABI 5-like protein 5	1.53	0.000
In Y7-1 under control as control (Y7-1) vs. control (Y478)			
Zm00001d018178	ABI 5-like protein 5	− 1.33	0.000
In Y478 under moderate drought as drought (Y478) vs. under control (Y478)			
Zm00001d050018	ABI 5-like protein 5	1.81	0.000
Zm00001d031790	ABI 5-like protein 5	1.11	0.000
Zm00001d018178	ABI 5-like protein 5	1.15	0.000
In Y478 under moderate drought-exogenous ABA as moderate drought-exogenous ABA (Y478) vs. control -exogenous ABA (Y478)			
Zm00001d018178	ABI 5-like protein 5	1.57	0.000
Zm00001d031790	ABI 5-like protein 5	1.23	0.000
Zm00001d050018	ABI 5-like protein 5	1.69	0.000

Foliar spraying with ABA started on the first day when the pot mix moisture was at the upper threshold of 50% for moderate drought.The results were based on transcriptome sequencing of the second fully-expanded leaves down from the top of 3 individual plants 9 d after the pot mix moisture was at the upper threshold of 50% for moderate drought.

ABA, Abscisic acid; ABI, ABA-insensitive; DEG, Differentially expressed gene; GA, Gibberellin; padj: Adjust *p*-value; T4, Transgenic generation 4; Y478, Maize inbred line Ye478; Y7-1, Y478 overexpressing *ZmPMP3g.*

^a^Positive and negative values indicated up-regulation and down-regulation of gene expression, respectively.

## Materials and methods

### Maize and *ZmPMP3g*

Maize lines used were HZ4, C7-2, Y478 and Z58. The maize gene *ZmPMP3g* was cloned by our laboratory^13^.

### Pot mix preparation

In brief, the tillage soil from the experimental field of Guangxi University was collected, sun-dried, sieved, and then fully mixed with the local commercial organic fertilizer (8:2/w:w). The prepared mix was loaded in pots (31 cm in diameter and 21 cm in height), 5 kg per pot. The pot mix was saturated with tap water, monitored for moisture by using the Dong mei DT001 soil tester (Shanghai, China) equipped with a thin and long detector following the instructions of the manufacturer, and then started either transplanting plants or sowing maize seeds when the moisture of the pot mix about 1 cm below the surface of the pot mix reached 70%.

### *ZmPMP3g* gene transfer, and Basta screening of transformants

The *ZmPMP3g* cDNA ranging from start codon to stop codon was cloned into *NcoI* and *BamH*I restriction sites downstream of the 3×CaMV35S promoter of plasmid pGSA1252 of Basta resistance gene, generating an expression construct of pGSA1252::*ZmPMP3g*. The construct was introduced into maize lines thorugh *Agrobacterium tumefaciens* strain LBA4404 mediated infection of the mature seed embryos. The LBA4404-infected maize embryos were transferred onto Murashige and Skoog medium plates containing 0.1 mg/L IAA, 250 mg/L cefotaxime, 20 ppm herbicide Basta and 30 g/L sucrose, and then allowed to grow for 7 d at 28 °C under a cycle of light/16 h and dark/8 h. The Basta-resistant maize plantlets were transplanted into sandy soil in trays and then grew for 10 d at room temperature followed by foliar spraying with 40 ppm Basta once. After 7 days of spraying with Basta, the maize seedlings that survived were transferred into the pot mix and grew in the glass greenhouse with natural lighting and air humidity conditions.

### Identification of transfomants by PCR and Southern blotting

The genomic DNA was isolated from maize leaves by using the Plant Genomic DNA Kit (Beijing ComWin Biotech Co., Ltd. China). PCR was performed in a 20-μL reaction system containing genomic DNA template, DNA sequence-specific primers and 2× Es Taq Master Mixture (Beijing ComWin Biotech Co., Ltd. China). The primers used were listed in Table 5. The PCR-amplified DNA was further verified by sequencing.

**Table 5:** The primers used in PCR and qPCR.

Used in	Primer	5′ → 3′
PCR	Bar-F	GCACCATCGTCAACCACTACATCG
	Bar-R	AAATCTCGGTGACGGGCAGGAC
	35S-F	CGTCTTCAAAGCAAGTGGATTG
	ZmPMP3g-R	GCGGTACCGTGACAGATGATGCATGGGG
qPCR	ZmPMP3g-F	TCTACGCCATCTACGCCATC
	ZmPMP3g-R	TCAAGCACTACATACAGCACAAG
	Actin-F (for interner control actin gene)	TGCCCTTCCTCATGCTATTCTACG
	Actin-R (for interner control actin gene)	GGCGGAGGTTGTGAAGGAAT

Southern blotting was conducted as the conventional method. In brief, the genomic DNA was digested with *Hind*III. The DNA was amplified by PCR from the pGSA1252::*ZmPMP3g* with primers of 35S-F and ZmPMP3g-R (Table 5), and then labelled as DNA probe by using the DIG High Prime DNA Labelling and Detection Starter Kit II (Roche, Sweden).

### Analysis of *ZmPMP3g* expression by qPCR

The total RNA was isolated by using the TransZol Up Plus RNA Kit (TransGen Biotech, Beijing, China). The first-strand cDNA was synthesized with total RNA by using the PrimeScript™ RT reagent kit containing the gDNA Eraser (TaKaRa, Dalian, China). The first-strand cDNA product was diluted 10 times and then used for qPCR. The qPCR was performed in a 10-μL reaction system containing 2 µL of the 10 times-diluted cDNA product and sequence-specific primers by using the SYBR^®^ Premix Ex Taq™ II (Tli RNaseH Plus) kit (TaKaRa). The qPCR instrument used was an ABI StepOne Plus™ Real-Time PCR System (Applied Biosystem, Temecula, CA, US). The interner control gene was maize actin gene (GenBamk accession no. XM_008650452.3). The primers used were listed in Table 5. The gene expression level in each maize line under each treatment was estimated as 2^−ΔCt [Ct (*ZmPMP3g*) − Ct (*actin*)]^ in the same maize line under the same treatment.

### Pot experiments

Mazie seeds were sowed in the pot mix with 70% moisture and grew in the glass greenhouse with conditions indicated above. After emergence, maize seedlings of health and uniform growth were left. During maize growth, the pot mix moisture was monitored daily as mentioned above. If the moisture of the pot mix about 1 cm below the surface of the pot mix was lower than 70%, the pot mix was sprayed with water. When maize seedlings grew to the 5-leaf stage, the pot mix of another group of seedlings was treated for drought by withholding water. Drought degree  was determined according to the moisture of the pot mix about 5 cm below the surface of the pot mix, which was defined by reference to the literature^51^ and classified into moderate drought of ≤ 50% a mix moisture ≥ 40% and severe drought of < 40% a mix moisture > 30%.

As for the combined treatments of drought/control and ABA, when the pot mix was in the first day of moderate and severe drought, respectively, the maize plants were treated by foilia spraying with 15 mg/L ABA (Sigma, USA) as described by^11^ while the seedlings in control treatment were sprayed with an equal volume of tap water.

Total 15 pots were set for each maize line under each treatment. The dates for analyses after treatments were somewhat different according to the specific situation, which were stated in the figure legends and footnotes. However, the tissues were sampled at 10:00 a.m., and/or immediately frozen in liquid nitrogen for analyses.

### Leaf wilting index analysis

Calculation of leaf wilting index was based on leaf wilting grading standards pre-established by our laboratory. Leaf wilting grade standards were level 0 (no wilting), level I (leaf drooping and slightly wilting), level II (partially shrivelled), level III (whole leaf shrivelled), and level IV (dried or died). The leaf wilting index was then calculated as a formular of [(the number of leaves of level 0 × 0) + (the number of leaves of level I × 1) + (the number of leaves of level II × 2) + (the number of leaves of level III × 3) + (the number of leaves of level IV × 4)]/(the total number of counted leaves  × 4).

### Assay of RWC

The RWC was assayed as described by^52^ with modifications. In brief, 1 g of fresh fully-expanded leaves was cut into small pieces and immersed for 80 min in distilled water. The immersed leaf pieces were collected and then weighed (saturated fresh weight, SFW). After then, the immersed leaf pieces were oven dried to constant weight at 80 °C and then weighed (dry weight, DW). RWC [%] was calculated as a formular of (1 − DW)/(SFW − DW) × 100.

### Assay of LWP

In brief, the surface of fresh fully-expanded leaves was lightly polished by using sandpaper until the leaf mesophyll was exposed. The polished leaves were then immediately detected for LWP by using the WP4-T Dewpoint PotentiaMeteR (METER, US) according to the manufacturer's instructions.

### Measurement of total root length

The roots were first scanned by using the Epson Expression 11000XL scanner (Japan) under 1200 dpi and then further analyzed for total root length by using the RhizoVisionExplorer-2.0.3 software of the WinRHIZO root analysis system WinRHIZO software (Regent Instruments Canada Inc. Canada) under built-in default parameters according to the instructions of the manufacturer.

### Assay of osmoprotectants

The betaine in the tissues was measured following the high-performance liquid chromatography (HPLC) method^53^ but with modifications. Briefly, 1 g of frozen tissues was homogenized in 2 mL of pre-cooled pure methanol by using the scientz-192 tissue grinder (Ningbo, China), The resulting homogenate was diluted to 50 time with Millipore ultra-pure water, concentrated and extracted for 1 h at 75 °C by using a BUCHI R-215 rotary evaporator (Switzerland), cooled on ice, and then filtered with a 0.45-µm filter membrane. The filtrate was then concentrated to a thick paste at 60 °C. The paste was dissolved in 2 mL of ultra-pure water, and filtered. The filtrate was then treated for 30 s by ultrasonic degassing. The ultrasonic degassing-treated filtrate was used for betaine analysis by using the Waters e2695 Alliance HPLC system equipped with a Waters 2998 photodiode array detector and the Empower 3.0 software. The internal standard used was analytical pure grade betaine.

The soluble sugars in the tissues were measured following the HPLC-evaporative light-scattering detector method^54^ but with modifications. Briefly, 0.1 g of frozen tissues was homogenized in 2 mL of ultra-pure water by using the grinder, and extracted in 80 °C water bath through oscillation for 20 min. The extract was centrifuged at 8000 rpm for 10 min. The resulting supernatant was transferred into a volumetric flask, diluted to 10 mL with ultra-pure water, filtered, and then treated for 30 s by ultrasonic degassing. The ultrasonic degassing-treated filtrate was used for total sugar analysis by using the Waters e2695 Alliance HPLC system equipped with a Waters 2424 evaporative light scattering detector and the Empower 3.0 software. The internal standard used was analytical pure grade d-fructose, d-glucose, and sucrose.

### Analysis of MDA

The MDA content in the tissues was determined according to the trichloroacetic acid (TCA)-thiobarbituric acid (TBA) method as described^55^ but with modifications. Briefly, 0.5 g of frozen tissue samples was fully homogenized in a pre-cooled mortar containing 10 mL of pre-cooled 10% TCA and a small amount of quartz sand on ice. The resulting homogenate was centrifuged at 4 °C and 12,000*g* for 10 min. The 1 mL of the supernatant resulting from centrifugation was mixed with 1 mL of 0.6% TBA, allowed to react in 100 °C water bath for 15 min, and then quickly cooled to room temperature on ice. The optical density (OD) values of the resulting reaction mixture at 450 nm, 532 nm, and 600 nm were measured by using the NanoDrop 2000c spectrophotometer (Thermo Scientific, USA), respectively. The MDA content in the reaction solution is calculated according to the formula of C (µmol/L) = [6.45 × (OD_532_-OD_600_) − (0.56 × OD_450_)], and then converted into the MDA content in the tissues as nM g^−1^ fresh weight (FW).

### Assay of O_2_^−·^ and H_2_O_2_

H_2_O_2_ was assayed according to the method described in the literature^56^ but with modifications. Frozen tissues (0.1 g) was fully homogenized in a pre-cooled mortar containing 1 mL of pre-cooled 0.1% TCA. The resulting homogenate was centrifuged at 12,000*g* for 30 min. The 0.5 mL of the resulting supernatant was fully mixed with 0.5 mL of 10 mM phosphate buffered saline (PBS) buffer (pH 7.8) and 1 mL of 1 M KI. The OD value of the supernatant was then read for estimation of H_2_O_2_ content at 360 nm by using the NanoDrop 2000c spectrophotometer. The content of H_2_O_2_ was calculated against a calibration curve plotted with H_2_O_2_ standard, and then converted into the H_2_O_2_ content in the tissues as μg g^−1^ FW.

O_2_^−·^ was measured following the method described in the literature^57^ but with modifications. Frozen tissues (0. 2 g) was fully homogenized in a pre-cooled mortar containing 2 mL of pre-cooled PBS (pH 7.8). The resulting homogenate was centrifuged at 15,000*g* for 10 min. Four tubes were numbered 0–3 (No. 0–3), of which No. 1–3 tubes were added with 0.5 mL of the resulting homogenate respectively, and No.0 tube was added with 0.5 mL of distilled water. Then, 0.5 mL of 50 mM PBS and 1 mL of 1 mM hydroxylamine hydrochloride were added to each tube. The tubes were placed in 100 ºC water bath for 1 h, and then quickly cooled to room temperature on ice. Each tube was then added with 1 mL of 17 mM p-aminobenzene sulfonic acid and 1 mL of 7 mM α-naphthylamine, and placed in 25 °C water bath for 25 min. The OD value of the resulting reaction mixture in each tube was measured for estimation of O_2_^−·^ content at 530 nm by using the NanoDrop 2000c spectrophotometer. The content of O_2_^−·^ in the solution was calculated against the NO^2−^ concentration in a calibration curve plotted with sodium nitrite satandads and then converted into the H_2_O_2_ content in the tissues as μM g^−1^ FW.

### Analysis of activities of SOD and CAT enzymes

For each sample, 0.2 g of the tissues was homogenized in 5 mL of 50 mM pre-cooled PBS (pH 7.8), transfered to a tube and then centrifuged for 20 min at 12,000*g* at 4 °C. The resulting supernatant was used as the crude proteinase extract for analyses of SOD and CAT activity.

The SOD activity was analyzed as the method described in the literature^58^ but with modifications. In brief, the reaction mixture was prepared with PBS (pH 7.8), which was composed of 1.3 μM riboflavin, 13 μM methionine, 63 μM NBT, 0.05 M sodium carbonate (pH 10.2). The 3 mL of reaction mixture were fully mixed with 30 μL of the extract. The mixture was then illuminated for 20 min under a light of 4000 lx in glass test tube. The glass test tubes that were added with both 3 mL reaction mixture and 30 μL PBS served as control. After illumination, the OD value of the resulting reaction mixture was measured for estimation of SOD activity at 530 nm in dark by using the NanoDrop 2000c spectrophotometer.

The CAT activity was analyzed as the method described in the literature^59^ but with modifications. In brief, the reaction mixture was composed of 457.2 mL of 0.15 mM PBS (pH 7.0) and 0.3092 mL of 30% H_2_O_2_. The 3 mL of reaction mixture were fully mixed with 30 μL of the extract, immediately read for OD values once at 240 nm every 30 s for total 5 min. The decrease of OD _240_ value by 0.01 per minute was defined as one enzyme activity unit (U).

### Analysis of endogenous ABA and GA

The ABA and GA content in the tissues was detected by MetWare (http://www.metware.cn/) based on the AB Sciex QTRAP 6500 LC–MS/MS platform following the methods decribed in the literature^60^ and conducted as the in-house protocols provided by the Genedenovo Biotechnology Co., Ltd (Guangzhou, China). In brief, the frozen tissue samples were ground to powder by using the MM400 ball mill (Retsch, Germany) at 30 Hz for 1 min. The 50 mg of the powder were fully mixed with an appropriate amount of internal standard and 1 mL of methanol/water/formic acid (15:4:1, v/v/v). The resulting extraction solution was further concentrated by using the CentriVap vacuum concentrator (LABCONCO, US). The concentrate was re-dissolved with 100 μ L 80% methanol/water solution, and filtered with a 0.22-μm filter membrane for LC–MS/MS analysis. The LC–MS/MS analysis was conducted as the following conditions: 550 °C electrospray ionization, 5,500 V MS voltage in positive ion mode, − 4,500 V MS voltage in negative ion mode, and 35 psi curtain gas. In Q-Trap 6500+, each ion pair was scanned and detected according to the optimized de-clustering potential and collision energy.

### Transcriptome sequencing

Transcriptome sequencing was based on the sequencing platform of the Novogene (Beijing, China). In brief, RNA was isolated from the second fresh leaves down from tops of plants. RNA degradation and contamination were monitored on 1% agarose gels. RNA purity was checked by using the NanoPhotometer^®^ spectrophotometer (IMPLEN, CA, USA). RNA integrity was assessed by using a RNA Nano 6000 Assay Kit of the Bioanalyzer 2100 system. A total of 1 µg RNA for each sample was used for construction of sequencing libraries. The libraries were generated by using a NEBNext^®^ UltraTM RNA Library Prep Kit for Illumina^®^ (NEB, USA) and sequenced by using the Illumina Novaseq platform. The transcriptome for each maize line under each treatment was repeated with the second fully-expanded leaves from 3 individual plants. The data were analyzed based on the Novomagic cloud platform (https://magic.novogene.com/) (Novogene, Beijing, China). The DEGs were defined as a |log2 fold change| of ≥ 1 under a adjust *p*-value (padj) of < 0.05.

### Statistical analysis

The statistically significant differences between the data were determined by SPSS version 17.0 (http://www.spss.com/) at *p* < 0.05 or *p* < 0.01.

### Statement of plants involved in this research

The wild type maize materials used in this study were from the Institute of Crop Sciences, CAAS, China, which comply with relevant institutional, national, and international guidelines and legislation in China.

## Supplementary Information

Supplementary Figure S1.

Supplementary Figure S2.

Supplementary Figure S2.

Supplementary Figure S3.

Supplementary Table S1.

Supplementary Table S2.

Supplementary Table S3.

## Data Availability

All data are available in the text and supplementary data without undue reservation. The transcriptome datasets generated and/or analyzed during the current study are available in the NCBI’s GEO repository under accession number GSE220124 (https://www.ncbi.nlm.nih.gov/geo/info/linking.html).

## References

[1] Rocha PS (2016). Plant abiotic stress-related *RCI2/PMP3s*: Multigenes for multiple roles. Planta.

[2] Kim HS, Park W, Lee HS, Shin JH, Ahn SJ (2021). Subcellular journey of rare cold inducible 2 protein in plant under stressful condition. Front. Plant Sci..

[3] Zhang D (2022). Overexpression of MsRCI2D and MsRCI2E enhances salt tolerance in alfalfa *(Medicago sativa* L.) by stabilizing antioxidant activity and regulating ion homeostasis. Int. J. Mol. Sci..

[4] Kim HS (2022). CsRCI2D enhances high-temperature stress tolerance in *Camelina sativa* L. through endo-membrane trafficking from the plasma membrane. Plant Sci..

[5] Inada M, Ueda A, Shi W, Takabe T (2005). A stress inducible plasma membrane protein 3 (AcPMP3) in a monocotyledonous halophyte, *Aneurolepidium chinense*, regulates cellular Na^+^ and K^+^ accumulation under salt stress. Planta.

[6] Ben Romdhane W (2017). Ectopic expression of *Aeluropus littoralis* plasma membrane protein gene *AlTMP1* confers abiotic stress tolerance in transgenic tobacco by improving water status and cation homeostasis. Int. J. Mol. Sci..

[7] Medina J, Ballesteros ML, Salinas J (2007). Phylogenetic and functional analysis of Arabidopsis RCI2 genes. J. Exp. Bot..

[8] Li C (2021). Overexpression of MsRCI2A, MsRCI2B, and MsRCI2C in Alfalfa (*Medicago sativa* L.) provides different extents of enhanced alkali and salt tolerance due to functional specialization of MsRCI2s. Front. Plant Sci..

[9] Kumar P (2022). Salinity stress tolerance and omics approaches: Revisiting the progress and achievements in major cereal crops. Heredity.

[10] Malenica N, Dunić JA, Vukadinović L, Cesar V, Šimić D (2021). Genetic approaches to enhance multiple stress tolerance in maize. Genes.

[11] Fu J (2012). Isolation and characterization of maize *PMP3* genes involved in salt stress tolerance. PLoS ONE.

[12] Zhao Y (2014). Identification and characterization of the *RCI2* gene family in maize (*Zea mays*). J. Genet..

[13] Qing DJ (2009). Comparative profiles of gene expression in leaves and roots of maize seedlings under conditions of salt stress and the removal of salt stress. Plant Cell Physiol..

[14] Li Y, Wang TY (2010). Germplasm base of maize breeding in China and formation of foundation parents. J. Maize Sci..

[15] Cassab GI, Eapen D, Campos ME (2013). Root hydrotropism: An update. Am. J. Bot..

[16] Rontein D, Basset G, Hanson AD (2002). Metabolic engineering of osmoprotectant accumulation in plants. Metab. Eng..

[17] Rawyler A, Arpagaus S, Braendle R (2002). Impact of oxygen stress and energy availability on membrane stability of plant cells. Ann. Bot..

[18] Catala A (2011). Lipid peroxidation of membrane phospholipids in the vertebrate retina. Front. Biosci..

[19] Birben E, Sahiner UM, Sackesen C, Erzurum S, Kalayci O (2012). Oxidative stress and antioxidant defense. World Allergy Organ. J..

[20] Carocho M, Ferreira IC (2013). A review on antioxidants, prooxidants and related controversy: Natural and synthetic compounds, screening and analysis methodologies and future perspectives. Food Chem. Toxicol..

[21] Agarwal PK, Jha B (2010). Transcription factors in plants and ABA dependent and independent abiotic stress signaling. Boliogical Plantarum.

[22] Sharp RE, LeNoble ME, Else MA, Thorne ET, Gherardi F (2000). Endogenous ABA maintains shoot growth in tomato independently of effects on plant water balance: Evidence for an interaction with ethylene. J. Exp. Bot..

[23] Sobeih WY, Dodd IC, Bacon MA, Grierson D, Davies WJ (2004). Long-distance signals regulating stomatal conductance and leaf growth in tomato (*Lycopersicon esculentum*) plants subjected to partial root-zone drying. J. Exp. Bot..

[24] Dietz KJ, Vogel MO, Viehhauser A (2010). AP2/EREBP transcription factors are part of gene regulatory networks and integrate metabolic, hormonal and environmental signals in stress acclimation and retrograde signalling. Protoplasma.

[25] Wilkinson S, Davies WJ (2010). Drought, ozone, ABA and ethylene: New insights from cell to plant to community. Plant Cell Environ..

[26] Feller A, Machemer K, Braun EL, Grotewold E (2011). Evolutionary and comparative analysis of MYB and bHLH plant transcription factors. Plant J..

[27] Huang GT (2012). Signal transduction during cold, salt, and drought stresses in plants. Mol. Biol. Rep..

[28] Nuruzzaman M, Sharoni AM, Kikuchi S (2013). Roles of NAC transcription factors in the regulation of biotic and abiotic stress responses in plants. Front. Microbiol..

[29] Roychoudhury A, Paul S, Basu S (2013). Cross-talk between abscisic acid-dependent and abscisic acid-independent pathways during abiotic stress. Plant Cell Rep..

[30] Yoshida T, Mogami J, Yamaguchi-Shinozaki K (2014). ABA-dependent and ABA-independent signaling in response to osmotic stress in plants. Curr. Opin. Plant Biol..

[31] Kapoor K (2018). Phytoglobins regulate nitric oxide-dependent abscisic acid synthesis and ethylene-induced program cell death in developing maize somatic embryos. Planta.

[32] Liu S, Lv Z, Liu Y, Li L, Zhang L (2018). Network analysis of ABA-dependent and ABA-independent drought responsive genes in *Arabidopsis thaliana*. Genet. Mol. Biol..

[33] Wang YG (2018). Interaction network of core ABA signaling components in maize. Plant Mol. Biol..

[34] Sah SK, Reddy KR, Li J (2016). Abscisic acid and abiotic stress tolerance in crop plants. Front. Plant Sci..

[35] Liu Y (2019). MYC2 regulates the termination of jasmonate signaling via an autoregulatory negative feedback loop. Plant Cell.

[36] Schwechheimer C (2008). Understanding gibberellic acid signaling–are we there yet?. Curr. Opin. Plant Biol..

[37] Kim GB, Son SU, Yu HJ, Mun JH (2019). *MtGA2ox10* encoding C20-GA2-oxidase regulates rhizobial infection and nodule development in *Medicago truncatula*. Sci. Rep..

[38] Blanco-Touriñán N, Serrano-Mislata A, Alabadí D (2020). Regulation of DELLA proteins by post-translational modifications. Plant Cell Physiol..

[39] Koornneef M, Bentsink L, Hilhorst H (2002). Seed dormancy and germination. Curr. Opin. Plant Biol..

[40] Mutasa-Göttgens E, Hedden P (2009). Gibberellin as a factor in floral regulatory networks. J. Exp. Bot..

[41] Davière JM, Achard P (2016). A pivotal role of DELLAs in regulating multiple hormone signals. Mol. Plant.

[42] Conti L (2017). Hormonal control of the floral transition: Can one catch them all ?. Dev. Boil..

[43] Zhang HJ (2014). Melatonin promotes seed germination under high salinity by regulating antioxidant systems, ABA and GA_4_ interaction in cucumber (*Cucumis sativus* L.). J. Pineal Res..

[44] Verma V, Ravindran P, Kumar PP (2016). Plant hormone-mediated regulation of stress responses. BMC Plant Biol..

[45] Cheng M (2022). H_2_O_2_ and Ca^2+^ signaling crosstalk counteracts ABA to induce seed germination. Antioxidants.

[46] Zhang ZL (2009). A negative regulator encoded by a rice *WRKY* gene represses both abscisic acid and gibberellins signaling in aleurone cells. Plant Mol. Biol..

[47] Shu K (2016). ABI4 mediates antagonistic effects of abscisic acid and gibberellins at transcript and protein levels. Plant J..

[48] Kang X (2018). HRB2 and BBX21 interaction modulates *Arabidopsis ABI5* locus and stomatal aperture. Plant Cell Environ..

[49] Yang X, Bai Y, Shang J, Xin R, Tang W (2016). The antagonistic regulation of abscisic acid-inhibited root growth by brassinosteroids is partially mediated via direct suppression of ABSCISIC ACID INSENSITIVE 5 expression by BRASSINAZOLE RESISTANT 1. Plant Cell Environ..

[50] Jiang G (2014). Regulation of inflorescence branch development in rice through a novel pathway involving the pentatricopeptide repeat protein sped1-D. Genetics.

[51] Zhang Q, Zou XK, Xiao FJ (2006). Classification of meteorologic category, GB/T 20481–2006.

[52] Phookaew P, Netrphan S, Sojikul P, Narangajavana J (2014). Involvement of miR164- and miR167-mediated target gene expressions in responses to water deficit in cassava. Biol. Plant..

[53] Liu ZG, Tao YD, Shao Y, Zhang HG (2012). Determination of betaine in *Lycium ruthenicum* Murr. and L*ycium barbarum *L. Chin. J. Spectrosc. Lab..

[54] Xiu L, Liu JS, Cai D, Zheng MZ (2011). Determination of soluble sugar content in fresh corn. Food Sci..

[55] Zhao SJ, Xu C, Zou Q, Meng Q (1994). Improvements of method for measurement of malondialdehyde in plant tissues. Plant Physiol. Commun..

[56] Ryan A, Cojocariu C, Possell M, Davies WJ, Hewitt CN (2009). Defining hybrid poplar (*Populus deltoides* x *Populus trichocarpa*) tolerance to ozone: Identifying key parameters. Plant Cell Environ..

[57] Elstner EF, Heupel A (1976). Inhibition of nitrite formation from hydroxylammoniumchloride: A simple assay for superoxide dismutase. Anal. Biochem..

[58] Giannopolitis CN, Ries SK (1997). Superoxide dismutases. I. Occurrence in higher plants. Plant Physiol..

[59] Aebi H (1984). Catalase in vitro. Methods Enzymol..

[60] Li Y, Zhou C, Yan X, Zhang J, Xu J (2016). Simultaneous analysis of ten phytohormones in *Sargassum horneri* by high-performance liquid chromatography with electrospray ionization tandem mass spectrometry. J. Sep. Sci..

[61] Chen S (2016). Identification and characterization of tomato gibberellin 2-oxidases (GA2oxs) and effects of fruit-specific *SlGA2ox1* overexpression on fruit and seed growth and development. Hortic. Res..

[62] Katyayini NU, Rinne PLH, Tarkowská D, Strnad M, van der Schoot C (2020). Dual role of gibberellin in perennial shoot branching: Inhibition and activation. Front. Plant Sci..

[63] Lo SF (2008). A novel class of gibberellin 2-oxidases control semidwarfism, tillering, and root development in rice. Plant Cell.

[64] Zentella R (2007). Global analysis of della direct targets in early gibberellin signaling in *Arabidopsis*. Plant Cell.

[65] Gou J (2018). From model to crop: Functional characterization of *SPL8* in *M. truncatula* led to genetic improvement of biomass yield and abiotic stress tolerance in alfalfa. Plant Biotechnol. J..

