# Supplementary figures and images for: Characterization of ZmPMP3g function in drought tolerance of maize
Source: Sci Rep. 2023 May 5;13:7375. doi: 10.1038/s41598-023-32989-4 (PMC10163268; doi:10.1038/s41598-023-32989-4)

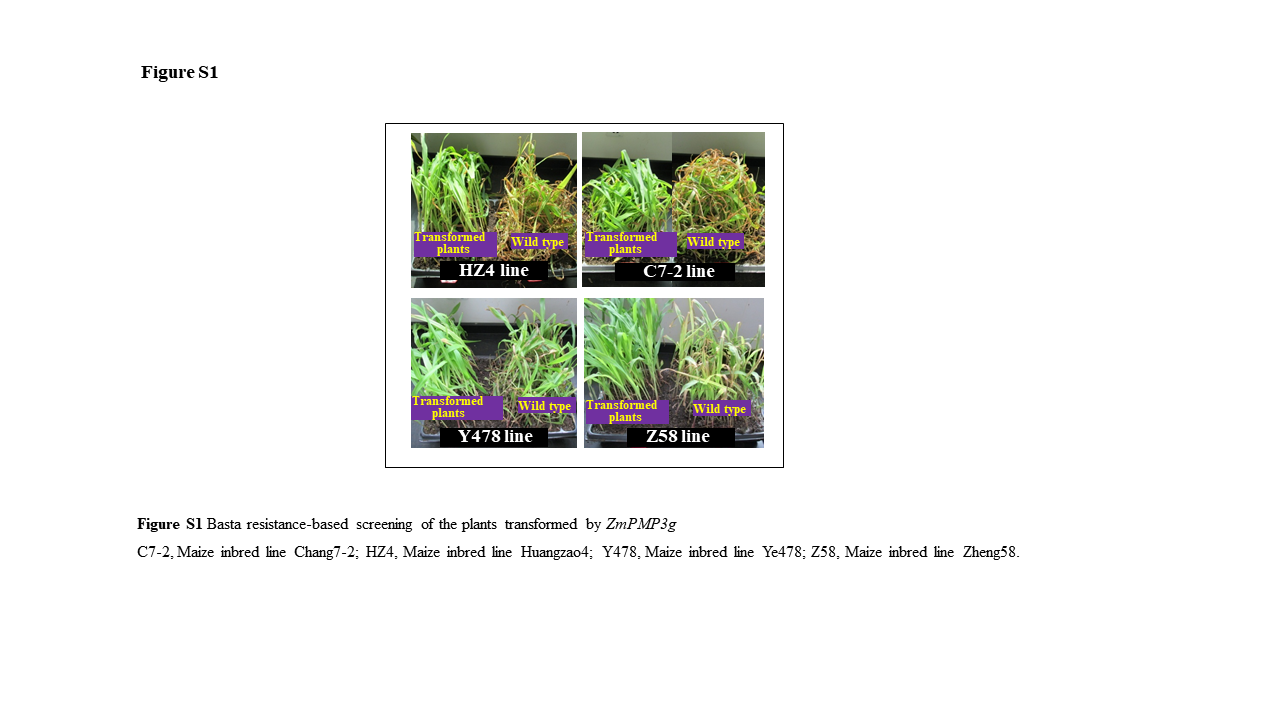

Supplement: Supplementary file 1 — Supplementary Figure S1. [file 41598_2023_32989_MOESM1_ESM.tif]

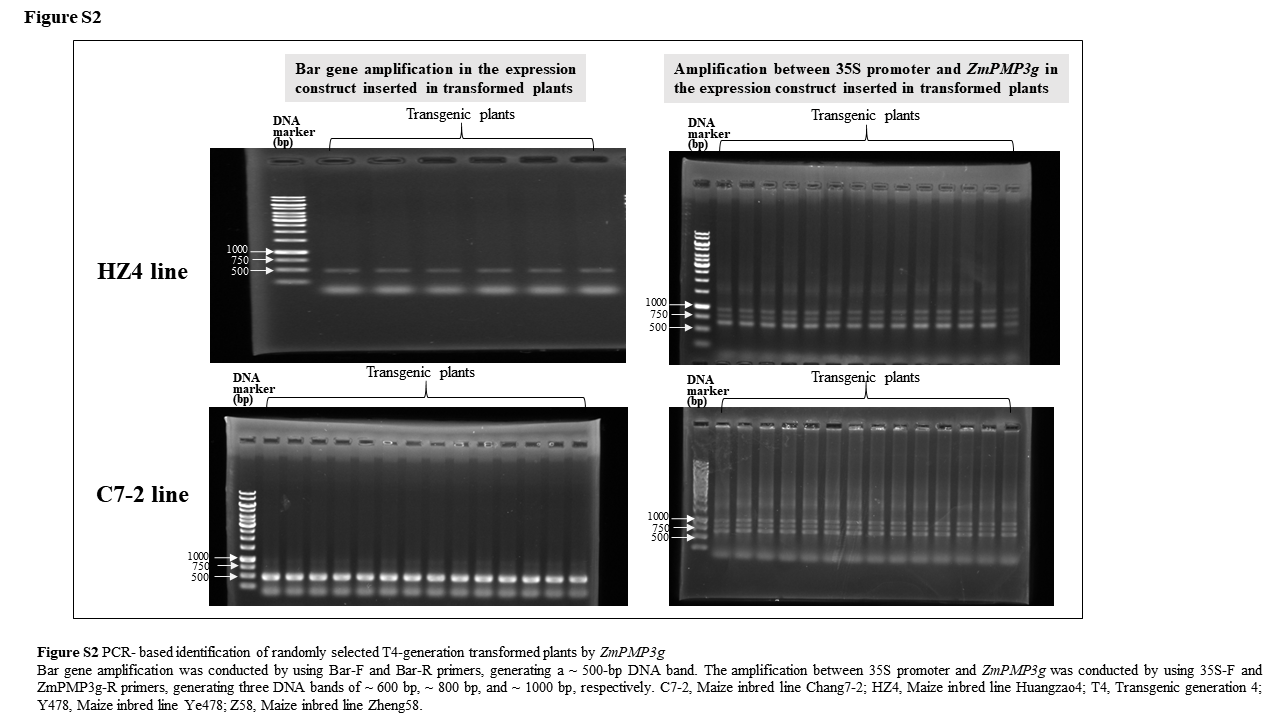

Supplement: Supplementary file 2 — Supplementary Figure S2. [file 41598_2023_32989_MOESM2_ESM.tif]

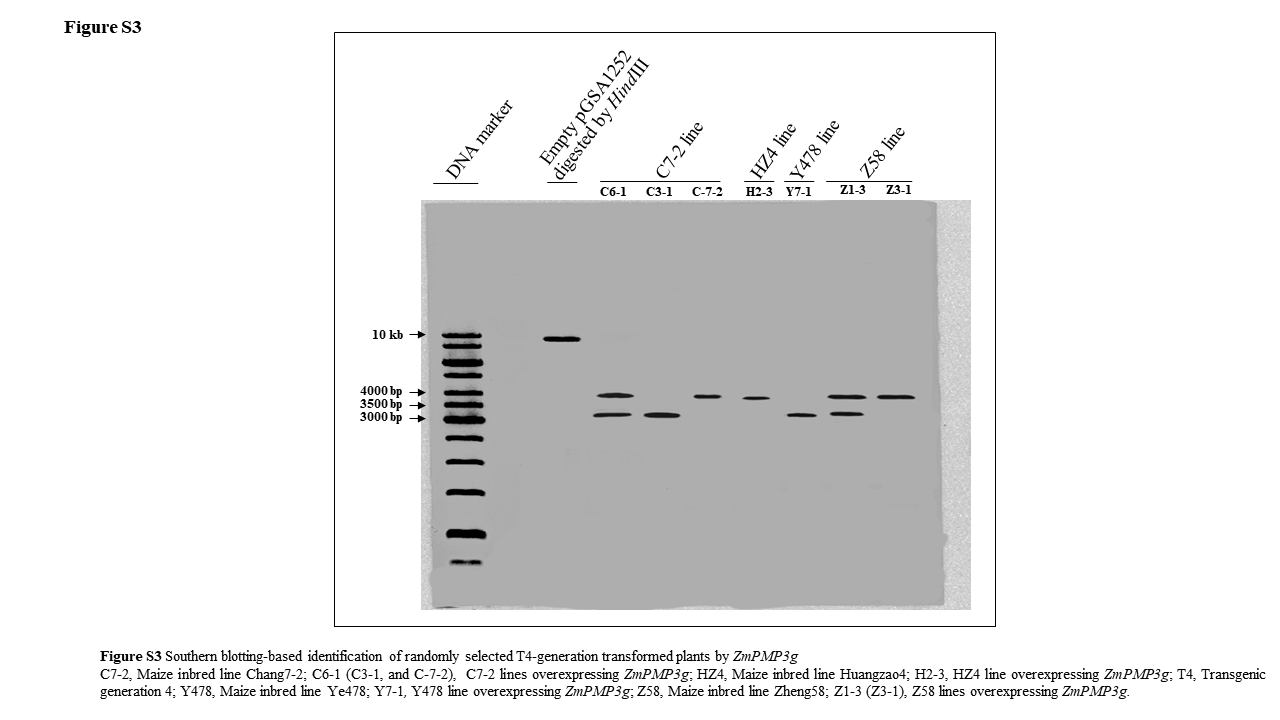

Supplement: Supplementary file 3 — Supplementary Figure S2. [file 41598_2023_32989_MOESM3_ESM.tif]

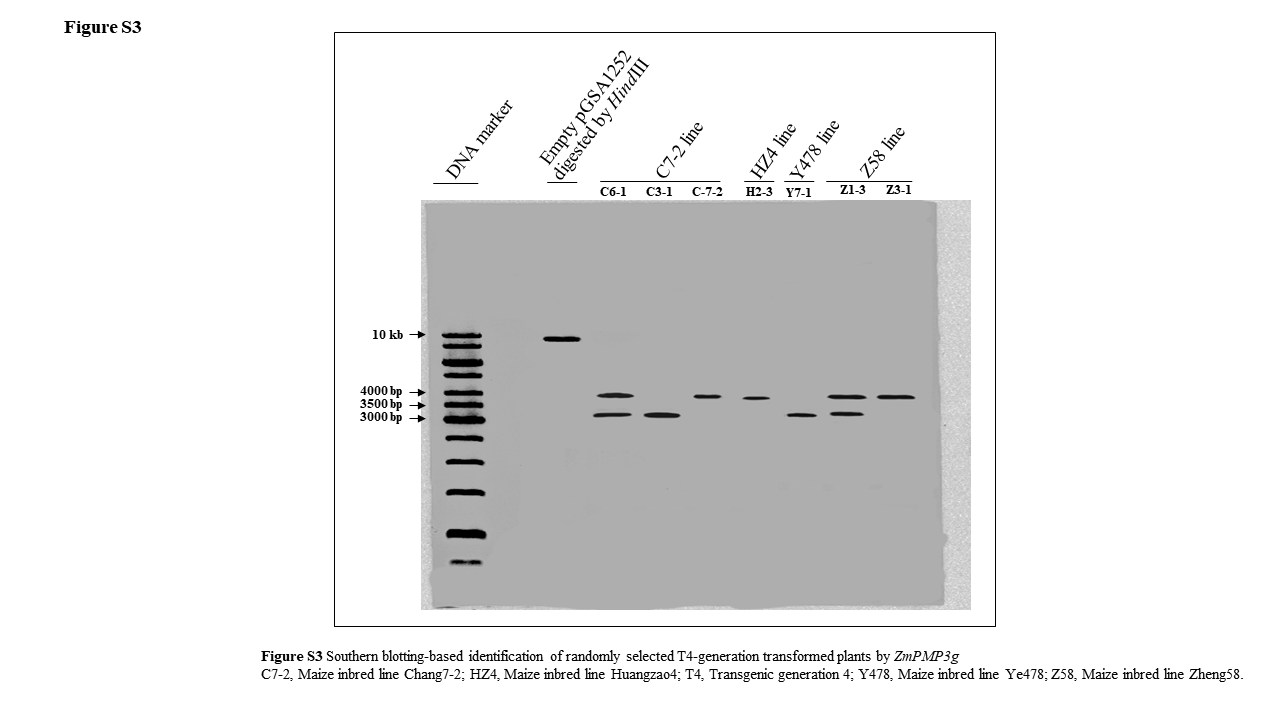

Supplement: Supplementary file 4 — Supplementary Figure S3. [file 41598_2023_32989_MOESM4_ESM.tif]
