# Supplementary material for: Characterization of ZmPMP3g function in drought tolerance of maize
Source: Sci Rep. 2023 May 5;13:7375. doi: 10.1038/s41598-023-32989-4 (PMC10163268; doi:10.1038/s41598-023-32989-4)
Supplement: Supplementary file 5 — Supplementary Table S1. [file 41598_2023_32989_MOESM5_ESM.docx]

| **Table S1** The DEGs in leaves of non-transgenic wild type Y478 under moderate drought alone as moderate drought (Y478) vs. control (Y478) in pot experiments with foliar spraying with exogenous ABA | | | | | |
| --- | --- | --- | --- | --- | --- |
| Gene ID |  | Gene description |  | log2 (fold change)^a^ | padj |
| Zm00001d022424 |  | NAC-transcription factor 122 |  | 6.56 | 0.000 |
| Zm00001d018819 |  | nine-cis-epoxycarotenoid dioxygenase5 |  | 5.37 | 0.000 |
| Zm00001d015664 |  | bZIP-transcription factor 36 |  | 5.29 | 0.011 |
| Zm00001d023294 |  | NAC-transcription factor 25 |  | 4.97 | 0.005 |
| Zm00001d010201 |  | MYB-transcription factor 25 |  | 4.06 | 0.000 |
| Zm00001d005750 |  | Transcription factor bHLH69 |  | 3.92 | 0.008 |
| Zm00001d045463 |  | NAC-transcription factor 86 |  | 3.64 | 0.000 |
| Zm00001d043729 |  | Transcription factor MYB86 |  | 3.28 | 0.000 |
| Zm00001d014405 |  | NAC-transcription factor 113 |  | 3.14 | 0.000 |
| Zm00001d033222 |  | viviparous14 |  | 3.09 | 0.000 |
| Zm00001d005208 |  | NAC-transcription factor 5 |  | 2.92 | 0.000 |
| Zm00001d028962 |  | WRKY-transcription factor 32 |  | 2.60 | 0.001 |
| Zm00001d042695 |  | SnRK2 serine threonine protein kinase 4 |  | 2.49 | 0.000 |
| Zm00001d039492 |  | myb-like transcription factor family protein |  | 2.49 | 0.000 |
| Zm00001d053220 |  | myb transcription factor 42 |  | 2.47 | 0.040 |
| Zm00001d046937 |  | bZIP-transcription factor 17 |  | 2.47 | 0.033 |
| Zm00001d000291 |  | bHLH-transcription factor 88 |  | 2.40 | 0.000 |
| Zm00001d010445 |  | Abscisic acid receptor PYL9 |  | 2.37 | 0.000 |
| Zm00001d042721 |  | bZIP-transcription factor 95 |  | 2.36 | 0.000 |
| Zm00001d005841 |  | bHLH-transcription factor 20 |  | 2.21 | 0.002 |
| Zm00001d041472 |  | NAC-transcription factor 108 |  | 2.14 | 0.000 |
| Zm00001d021086 |  | Transcription factor bHLH137 |  | 2.08 | 0.003 |
| Zm00001d040500 |  | bZIP-transcription factor 42 |  | 2.04 | 0.000 |
| Zm00001d038221 |  | NAC-transcription factor 20 |  | 1.99 | 0.001 |
| Zm00001d023529 |  | abscisic acid stress ripening1 |  | 1.98 | 0.000 |
| Zm00001d028999 |  | NAC-transcription factor 44 |  | 1.86 | 0.020 |
| Zm00001d002143 |  | bZIP-transcription factor 27 |  | 1.82 | 0.001 |
| Zm00001d050018 |  | bZIP-transcription factor 68 |  | 1.81 | 0.000 |
| Zm00001d044857 |  | AP2-EREBP-transcription factor 31 |  | 1.77 | 0.045 |
| Zm00001d030995 |  | bZIP-transcription factor 111 |  | 1.73 | 0.000 |
| Zm00001d001936 |  | G2-like-transcription factor 16 |  | 1.73 | 0.000 |
| Zm00001d004843 |  | abscisic acid stress ripening2 |  | 1.66 | 0.000 |
| Zm00001d034601 |  | NAC-transcription factor 49 |  | 1.58 | 0.000 |
| Zm00001d003712 |  | abscisic acid stress ripening3 |  | 1.57 | 0.000 |
| Zm00001d032024 |  | myb transcription factor38 |  | 1.53 | 0.000 |
| Zm00001d025864 |  | MYB-transcription factor 132 |  | 1.50 | 0.045 |
| Zm00001d050893 |  | NAC-transcription factor 40 |  | 1.49 | 0.000 |
| Zm00001d048901 |  | Transcription factor bHLH47 |  | 1.45 | 0.002 |
| Zm00001d049860 |  | NAC-transcription factor 75 |  | 1.41 | 0.009 |
| Zm00001d045044 |  | AP2-EREBP-transcription factor 160 |  | 1.38 | 0.000 |
| Zm00001d020025 |  | bZIP-transcription factor 9 |  | 1.27 | 0.001 |
| Zm00001d028984 |  | G2-like-transcription factor 26 |  | 1.23 | 0.000 |
| Zm00001d038585 |  | AP2-EREBP-transcription factor 196 |  | 1.16 | 0.000 |
| Zm00001d018178 |  | bZIP-transcription factor 4 |  | 1.15 | 0.000 |
| Zm00001d031790 |  | bZIP-transcription factor 49 |  | 1.11 | 0.000 |
| Zm00001d053162 |  | bZIP-transcription factor 110 |  | 1.04 | 0.013 |
| Zm00001d027929 |  | AP2-EREBP-transcription factor 182 |  | 1.04 | 0.010 |
| Zm00001d026271 |  | AP2-EREBP-transcription factor 205 |  | 1.04 | 0.002 |
| Zm00001d012404 |  | myb domain protein 3r-3 |  | 1.01 | 0.004 |
| Zm00001d042609 |  | NAC-transcription factor 109 |  | 1.01 | 0.046 |
| Zm00001d011969 |  | NAC-transcription factor 9 |  | -1.09 | 0.001 |
| Zm00001d018081 |  | AP2-EREBP-transcription factor 18 |  | -1.11 | 0.000 |
| Zm00001d033267 |  | bHLH-transcription factor 43 |  | -1.13 | 0.000 |
| Zm00001d003401 |  | general regulatory factor1 |  | -1.16 | 0.000 |
| Zm00001d003513 |  | zeaxanthin epoxidase1 |  | -1.17 | 0.003 |
| Zm00001d042288 |  | NAC-transcription factor 90 |  | -1.19 | 0.001 |
| Zm00001d010667 |  | bZIP-transcription factor 108 |  | -1.20 | 0.042 |
| Zm00001d051554 |  | abscisic acid 8'-hydroxylase2 |  | -1.22 | 0.006 |
| Zm00001d025544 |  | Zeaxanthin epoxidase chloroplastic |  | -1.27 | 0.000 |
| Zm00001d032694 |  | MYB-transcription factor 121 |  | -1.35 | 0.030 |
| Zm00001d016294 |  | Abscisic acid receptor PYL2 |  | -1.36 | 0.009 |
| Zm00001d044242 |  | bHLH-transcription factor 25 |  | -1.49 | 0.001 |
| Zm00001d028297 |  | bHLH-transcription factor 3 |  | -1.51 | 0.000 |
| Zm00001d040621 |  | Transcription factor bHLH69 |  | -1.60 | 0.000 |
| Zm00001d032032 |  | Transcription factor MYB86 |  | -1.65 | 0.011 |
| Zm00001d008968 |  | myb-like transcription factor family protein |  | -1.68 | 0.008 |
| Zm00001d041576 |  | Abscisic acid receptor PYL9 |  | -1.73 | 0.000 |
| Zm00001d018435 |  | Transcription factor bHLH137 |  | -1.77 | 0.026 |
| Zm00001d014701 |  | Transcription factor bHLH47 |  | -1.96 | 0.000 |
| Zm00001d007962 |  | myb domain protein 3r-3 |  | -1.99 | 0.000 |
| Zm00001d006065 |  | Abscisic acid receptor PYL2 |  | -2.11 | 0.010 |
| Zm00001d023411 |  | Myb family transcription factor PHL6 |  | -2.14 | 0.001 |
| Zm00001d028793 |  | Abscisic acid receptor PYL5 |  | -2.81 | 0.046 |
| Zm00001d032144 |  | AP2-EREBP-transcription factor 60 |  | -3.78 | 0.022 |
| The results were based on transcriptome sequencing of the second fully-expanded leaves down from the top of 3 individual plants 9 d after the pot mix moisture was at the upper threshold of 50% for moderate drought. ^a^ Positive and negative values indicated up-regulation and down-regulation of gene expression, respectively. ABA, Abscisic acid; DEG, Differentially expressed gene; padj: Adjust *p*-value; Y478, Maize inbred line Ye478. | | | | | |
