# Supplementary material for: Characterization of ZmPMP3g function in drought tolerance of maize
Source: Sci Rep. 2023 May 5;13:7375. doi: 10.1038/s41598-023-32989-4 (PMC10163268; doi:10.1038/s41598-023-32989-4)
Supplement: Supplementary file 6 — Supplementary Table S2. [file 41598_2023_32989_MOESM6_ESM.docx]

| **Table S2** The DEGs in leaves of non-transgenic wild type Y478 under a combination of moderate drought and exogenous ABA as a combination of moderate drought and exogenous ABA (Y478) vs. moderate drought (Y478) in pot experiments with foliar spraying with exogenous ABA | | | | | |  |
| --- | --- | --- | --- | --- | --- | --- |
| Gene ID | Gene description |  |  | log2 (fold change)^a^ | padj |  |
| Zm00001d052192 | Transcription factor bHLH99 |  |  | 7.54 | 0.001 |  |
| Zm00001d021298 | Probable glucan endo-13-beta-glucosidase BG4 |  |  | 4.71 | 0.012 |  |
| Zm00001d035621 | Aspartic proteinase A1 |  |  | 4.38 | 0.000 |  |
| Zm00001d014721 | Glycosyltransferase family 61 protein |  |  | 3.87 | 0.015 |  |
| novel.632 | - |  |  | 3.48 | 0.023 |  |
| Zm00001d047841 | 17.4 kDa class I heat shock protein |  |  | 3.35 | 0.005 |  |
| Zm00001d012584 | Putative AP2/EREBP transcription factor superfamily protein |  |  | 2.98 | 0.035 |  |
| novel.5434 | - |  |  | 2.92 | 0.023 |  |
| Zm00001d029405 | - |  |  | 2.86 | 0.036 |  |
| novel.2787 | - |  |  | 2.84 | 0.006 |  |
| Zm00001d007109 | Protein PIN-LIKES 7 |  |  | 2.72 | 0.048 |  |
| Zm00001d015225 | Vacuolar cation/proton exchanger 2 |  |  | 2.70 | 0.004 |  |
| Zm00001d026160 | Cortical cell-delineating protein%3B ZmGR1b protein |  |  | 2.49 | 0.007 |  |
| novel.4960 | - |  |  | 2.47 | 0.001 |  |
| Zm00001d014155 | 50S ribosomal protein L28 chloroplastic |  |  | 2.43 | 0.001 |  |
| Zm00001d009622 | Putative AP2/EREBP transcription factor superfamily protein |  |  | 2.30 | 0.001 |  |
| Zm00001d047542 | 17.6 kDa class II heat shock protein |  |  | 2.28 | 0.007 |  |
| Zm00001d047728 | Putative MAPKKK family protein kinase |  |  | 2.28 | 0.002 |  |
| Zm00001d020488 | Retrovirus-related Pol polyprotein LINE-1 |  |  | 2.24 | 0.016 |  |
| novel.3807 | PF06962:Putative rRNA methylase |  |  | 2.20 | 0.006 |  |
| Zm00001d002733 | Cyclin-U2-1 |  |  | 2.20 | 0.039 |  |
| Zm00001d039011 | Grx_I1-glutaredoxin subgroup III |  |  | 2.18 | 0.028 |  |
| Zm00001d039468 | Grx_A2-glutaredoxin subgroup III |  |  | 2.18 | 0.007 |  |
| Zm00001d016159 | prefoldin 6 |  |  | 2.14 | 0.001 |  |
| Zm00001d039935 | heat shock protein17.2 |  |  | 2.13 | 0.007 |  |
| Zm00001d012693 | Solute carrier family 23 member 2 |  |  | 2.12 | 0.033 |  |
| Zm00001d043290 | - |  |  | 2.11 | 0.001 |  |
| Zm00001d049364 | Ethylene-responsive transcription factor 4%3B Putative AP2/EREBP transcription factor superfamily protein |  |  | 2.03 | 0.005 |  |
| Zm00001d010540 | - |  |  | 2.02 | 0.037 |  |
| Zm00001d012171 | - |  |  | 2.02 | 0.003 |  |
| novel.874 | PF03732:Retrotransposon gag protein |  |  | 1.98 | 0.023 |  |
| Zm00001d052380 | scarecrow1 |  |  | 1.97 | 0.001 |  |
| Zm00001d011655 | MAP kinase kinase kinase18 |  |  | 1.94 | 0.028 |  |
| ENSRNA049472786 | Small nucleolar RNA Z188 |  |  | 1.92 | 0.003 |  |
| Zm00001d033987 | Heat stress transcription factor A-6b |  |  | 1.89 | 0.000 |  |
| Zm00001d014970 | - |  |  | 1.89 | 0.004 |  |
| Zm00001d009612 | DUF1645 family protein |  |  | 1.88 | 0.011 |  |
| Zm00001d010791 | Histidine-containing phosphotransfer protein 4 |  |  | 1.88 | 0.015 |  |
| Zm00001d037757 | Grx_C15-glutaredoxin subgroup III |  |  | 1.87 | 0.000 |  |
| Zm00001d011755 | Secreted protein |  |  | 1.84 | 0.014 |  |
| Zm00001d013109 | calcium-dependent protein kinase 10 |  |  | 1.84 | 0.000 |  |
| Zm00001d039842 | Disease resistance response protein-like; protein |  |  | 1.83 | 0.001 |  |
| Zm00001d024497 | ABC transporter G family member 14 |  |  | 1.81 | 0.001 |  |
| Zm00001d030498 | - |  |  | 1.81 | 0.038 |  |
| Zm00001d018107 | Protein EXORDIUM |  |  | 1.81 | 0.005 |  |
| Zm00001d018404 | Heparanase-like protein 3 |  |  | 1.80 | 0.001 |  |
| Zm00001d049218 | Two-pore potassium channel 1 |  |  | 1.78 | 0.010 |  |
| Zm00001d021978 | ARM repeat superfamily protein |  |  | 1.78 | 0.000 |  |
| Zm00001d042050 | Protein RETICULATA-RELATED 4 chloroplastic |  |  | 1.77 | 0.000 |  |
| Zm00001d025310 | Putative RING zinc finger domain superfamily protein |  |  | 1.75 | 0.000 |  |
| Zm00001d038164 | methyl binding domain |  |  | 1.71 | 0.002 |  |
| Zm00001d038806 | Chaperone protein ClpB1 |  |  | 1.71 | 0.000 |  |
| Zm00001d018298 | 17.4 kDa class III heat shock protein |  |  | 1.70 | 0.015 |  |
| Zm00001d046299 | HSF28 HSF type transcription factor |  |  | 1.69 | 0.046 |  |
| Zm00001d033857 | EPIDERMAL PATTERNING FACTOR-like protein 2 |  |  | 1.69 | 0.001 |  |
| Zm00001d003981 | Drought-induced protein 1 |  |  | 1.69 | 0.000 |  |
| Zm00001d008595 | Pirin-like protein 2 |  |  | 1.65 | 0.037 |  |
| Zm00001d049660 | ACR8 |  |  | 1.64 | 0.032 |  |
| Zm00001d008841 | 17.8 kDa class II heat shock protein |  |  | 1.64 | 0.000 |  |
| Zm00001d046888 | B-box zinc finger family protein%3B Putative B-box type zinc finger family protein |  |  | 1.62 | 0.003 |  |
| Zm00001d049240 | Alpha carbonic anhydrase 1 chloroplastic |  |  | 1.62 | 0.041 |  |
| Zm00001d050222 | - |  |  | 1.61 | 0.005 |  |
| Zm00001d003781 | Alpha carbonic anhydrase 1 chloroplastic |  |  | 1.60 | 0.019 |  |
| Zm00001d030339 | Peptidyl-prolyl cis-trans isomerase |  |  | 1.60 | 0.037 |  |
| Zm00001d017019 | ATFP4 |  |  | 1.58 | 0.030 |  |
| Zm00001d036676 | Putative B-box type zinc finger family protein |  |  | 1.57 | 0.000 |  |
| Zm00001d020686 | 1-aminocyclopropane-1-carboxylate oxidase2 |  |  | 1.56 | 0.005 |  |
| Zm00001d022307 | 10 kDa chaperonin |  |  | 1.56 | 0.000 |  |
| Zm00001d038067 | - |  |  | 1.55 | 0.001 |  |
| Zm00001d039910 | Protein ASPARTIC PROTEASE IN GUARD CELL 1 |  |  | 1.55 | 0.028 |  |
| Zm00001d034312 | - |  |  | 1.54 | 0.025 |  |
| Zm00001d044138 | siroheme uroporphyrinogen methyltransferase1 |  |  | 1.53 | 0.001 |  |
| Zm00001d047573 | F-box protein |  |  | 1.52 | 0.009 |  |
| Zm00001d005609 | protein phosphatase homolog7 |  |  | 1.52 | 0.019 |  |
| Zm00001d049347 | Zinc finger protein CONSTANS-LIKE 5 |  |  | 1.52 | 0.048 |  |
| Zm00001d052661 | - |  |  | 1.52 | 0.022 |  |
| Zm00001d014007 | senescence regulator |  |  | 1.51 | 0.010 |  |
| Zm00001d036306 | Putative RING zinc finger domain superfamily protein |  |  | 1.51 | 0.008 |  |
| Zm00001d052194 | 23.6 kDa heat shock protein mitochondrial |  |  | 1.50 | 0.005 |  |
| Zm00001d003006 | Aquaporin PIP2-5 |  |  | 1.49 | 0.001 |  |
| Zm00001d029806 | NEP1-interacting protein-like 1 |  |  | 1.49 | 0.039 |  |
| Zm00001d038291 | Ferredoxin-6%2C chloroplastic |  |  | 1.48 | 0.000 |  |
| Zm00001d049187 | phosphogluconate dehydrogenase3 |  |  | 1.48 | 0.005 |  |
| Zm00001d004357 | protein phosphatase homolog10 |  |  | 1.47 | 0.037 |  |
| Zm00001d039058 | DUF1645 family protein |  |  | 1.47 | 0.038 |  |
| Zm00001d002593 | Protein kinase |  |  | 1.46 | 0.011 |  |
| Zm00001d016850 | Molybdopterin synthase sulfur carrier subunit |  |  | 1.46 | 0.000 |  |
| Zm00001d013111 | DnaJ protein |  |  | 1.46 | 0.003 |  |
| Zm00001d038514 | Protein LURP-one-related 8 |  |  | 1.45 | 0.003 |  |
| Zm00001d046471 | Multiprotein-bridging factor 1c |  |  | 1.45 | 0.001 |  |
| Zm00001d052372 | Protein LURP-one-related 8 |  |  | 1.44 | 0.015 |  |
| Zm00001d018244 | - |  |  | 1.43 | 0.000 |  |
| Zm00001d050889 | Oxygen evolving enhancer protein 3 |  |  | 1.43 | 0.000 |  |
| Zm00001d025322 | - |  |  | 1.42 | 0.003 |  |
| Zm00001d039933 | 16.9 kDa class I heat shock protein 1 |  |  | 1.41 | 0.002 |  |
| Zm00001d026505 | - |  |  | 1.39 | 0.017 |  |
| Zm00001d002052 | Probable isoaspartyl peptidase/L-asparaginase 2 |  |  | 1.39 | 0.000 |  |
| Zm00001d011073 | AT5g02160 |  |  | 1.38 | 0.020 |  |
| Zm00001d028391 | Subtilisin-like protease SBT1.6 |  |  | 1.38 | 0.001 |  |
| Zm00001d044399 | photosystem II light harvesting complex gene B1B2 |  |  | 1.38 | 0.002 |  |
| Zm00001d038920 | DUF1639 family protein |  |  | 1.38 | 0.014 |  |
| Zm00001d034419 | Acyl-CoA N-acyltransferases (NAT) superfamily protein |  |  | 1.37 | 0.048 |  |
| Zm00001d027757 | Heat stress transcription factor A-6b |  |  | 1.36 | 0.025 |  |
| Zm00001d051362 | tonoplast intrinsic protein2 |  |  | 1.35 | 0.001 |  |
| Zm00001d041800 | BTB/POZ domain-containing protein |  |  | 1.35 | 0.021 |  |
| Zm00001d044728 | Class I heat shock protein 3 |  |  | 1.34 | 0.020 |  |
| Zm00001d010529 | Probable mediator of RNA polymerase II transcription subunit 37c |  |  | 1.33 | 0.000 |  |
| Zm00001d002562 | B3 domain-containing transcription factor NGA2 |  |  | 1.33 | 0.001 |  |
| Zm00001d045455 | Inositol-3-phosphate synthase isozyme 1 |  |  | 1.33 | 0.000 |  |
| Zm00001d039997 | NPR1 interactor |  |  | 1.33 | 0.001 |  |
| Zm00001d020623 | Copper transporter 5 |  |  | 1.33 | 0.016 |  |
| Zm00001d014386 | Patatin-like protein 6 |  |  | 1.33 | 0.012 |  |
| Zm00001d037275 | B12D protein |  |  | 1.32 | 0.048 |  |
| Zm00001d047260 | Whole genome shotgun sequence of line PN40024 scaffold_16.assembly12x (Fragment) |  |  | 1.32 | 0.010 |  |
| ZeamMr004 | rrn1818S ribosomal RNA |  |  | 1.32 | 0.001 |  |
| Zm00001d047883 | Probable carboxylesterase 17 |  |  | 1.31 | 0.010 |  |
| Zm00001d033369 | Gibberellin-regulated protein 1 |  |  | 1.31 | 0.008 |  |
| Zm00001d012626 | - |  |  | 1.30 | 0.047 |  |
| Zm00001d018161 | Ferredoxin--nitrite reductase chloroplastic |  |  | 1.30 | 0.034 |  |
| Zm00001d036441 | zinc finger (C2H2 type) family protein |  |  | 1.29 | 0.014 |  |
| Zm00001d021450 | Cytokinin riboside 5'-monophosphate phosphoribohydrolase LOG5 |  |  | 1.29 | 0.007 |  |
| Zm00001d038688 | Rho-related protein6 |  |  | 1.28 | 0.006 |  |
| Zm00001d042107 | Polcalcin Jun o 2 |  |  | 1.27 | 0.011 |  |
| Zm00001d005693 | - |  |  | 1.27 | 0.033 |  |
| Zm00001d035135 | Photosystem II core complex protein psbY%3B Photosystem II core complex proteins psbY |  |  | 1.26 | 0.003 |  |
| Zm00001d034527 | - |  |  | 1.25 | 0.001 |  |
| Zm00001d053528 | Cwf21 |  |  | 1.24 | 0.044 |  |
| Zm00001d051779 | Probable aspartyl protease |  |  | 1.24 | 0.001 |  |
| Zm00001d006547 | Histone H2A |  |  | 1.24 | 0.011 |  |
| Zm00001d027873 | - |  |  | 1.24 | 0.042 |  |
| Zm00001d033038 | Stress enhanced protein 1 chloroplastic |  |  | 1.23 | 0.031 |  |
| Zm00001d020898 | Heat shock protein 90-2 |  |  | 1.23 | 0.000 |  |
| Zm00001d027749 | Sulfate transporter 3.1 |  |  | 1.23 | 0.003 |  |
| Zm00001d009568 | Glutaredoxin-C9 |  |  | 1.23 | 0.035 |  |
| Zm00001d037095 | Putative RING zinc finger domain superfamily protein |  |  | 1.23 | 0.017 |  |
| Zm00001d021632 | - |  |  | 1.22 | 0.003 |  |
| Zm00001d011969 | NAC domain containing protein 84 |  |  | 1.22 | 0.004 |  |
| Zm00001d026271 | Putative AP2/EREBP transcription factor superfamily protein |  |  | 1.22 | 0.006 |  |
| Zm00001d033680 | dwarf plant8 |  |  | 1.21 | 0.002 |  |
| Zm00001d028670 | Photosynthetic NDH subunit of lumenal location 1 chloroplastic |  |  | 1.21 | 0.000 |  |
| Zm00001d044396 | Chlorophyll a-b binding protein 48%2C chloroplastic |  |  | 1.21 | 0.016 |  |
| Zm00001d039566 | 17.5 kDa class II heat shock protein |  |  | 1.21 | 0.000 |  |
| Zm00001d019510 | Folate/biopterin transporter family protein |  |  | 1.20 | 0.009 |  |
| Zm00001d022006 | Lipase-like protein |  |  | 1.20 | 0.014 |  |
| Zm00001d005945 | - |  |  | 1.20 | 0.044 |  |
| Zm00001d048313 | NAD(P)-linked oxidoreductase superfamily protein |  |  | 1.20 | 0.005 |  |
| Zm00001d009907 | alpha/beta-Hydrolases superfamily protein |  |  | 1.18 | 0.022 |  |
| Zm00001d021444 | Monooxygenase/ oxidoreductase |  |  | 1.18 | 0.027 |  |
| Zm00001d014755 | F-box/kelch-repeat protein SKIP25 |  |  | 1.18 | 0.029 |  |
| Zm00001d020670 | Homeobox-leucine zipper protein HAT3 |  |  | 1.17 | 0.010 |  |
| Zm00001d045125 | RING/U-box superfamily protein |  |  | 1.17 | 0.023 |  |
| Zm00001d015918 | - |  |  | 1.17 | 0.012 |  |
| Zm00001d018665 | LRR receptor-like serine/threonine-protein kinase RPK2 |  |  | 1.16 | 0.040 |  |
| ZeamMr002 | rrn2626S ribosomal RNA |  |  | 1.16 | 0.000 |  |
| Zm00001d012686 | - |  |  | 1.16 | 0.000 |  |
| Zm00001d010380 | Zinc finger CCCH domain-containing protein 23 |  |  | 1.16 | 0.004 |  |
| Zm00001d048795 | SPR1 |  |  | 1.15 | 0.037 |  |
| Zm00001d010759 | DNA-binding WRKY |  |  | 1.15 | 0.039 |  |
| Zm00001d028392 | actin depolymerizing factor5 |  |  | 1.15 | 0.005 |  |
| Zm00001d043240 | Sulfite exporter TauE/SafE family protein |  |  | 1.14 | 0.037 |  |
| Zm00001d032155 | CBL-interacting serine/threonine-protein kinase 15 |  |  | 1.14 | 0.000 |  |
| Zm00001d014606 | Peroxidase 45 |  |  | 1.14 | 0.037 |  |
| Zm00001d041332 | Zinc finger protein 7 |  |  | 1.14 | 0.000 |  |
| Zm00001d016943 | Photosynthetic NDH subunit of lumenal location 2 chloroplastic |  |  | 1.13 | 0.025 |  |
| Zm00001d053160 | - |  |  | 1.13 | 0.027 |  |
| Zm00001d033636 | Putative uncharacterized protein [Source:UniProtKB/TrEMBL;Acc:B6TAU5] |  |  | 1.13 | 0.007 |  |
| Zm00001d041965 | drought-induced 19 |  |  | 1.13 | 0.000 |  |
| Zm00001d019045 | Histone H2A |  |  | 1.13 | 0.014 |  |
| Zm00001d021422 | Histone H4 |  |  | 1.13 | 0.019 |  |
| Zm00001d031325 | 25.3 kDa heat shock protein chloroplastic |  |  | 1.13 | 0.005 |  |
| Zm00001d005421 | Aquaporin PIP2-2 |  |  | 1.12 | 0.000 |  |
| Zm00001d005029 | Protein SCARECROW |  |  | 1.12 | 0.001 |  |
| Zm00001d047060 | OSJNBb0016D16.16 protein; protein |  |  | 1.12 | 0.028 |  |
| Zm00001d022390 | Eukaryotic aspartyl protease family protein |  |  | 1.12 | 0.008 |  |
| Zm00001d053897 | Ubiquitin-conjugating enzyme E2 29 |  |  | 1.12 | 0.002 |  |
| Zm00001d022229 | Malate dehydrogenase chloroplastic |  |  | 1.11 | 0.004 |  |
| Zm00001d050837 | Gibberellin receptor GID1L2 |  |  | 1.11 | 0.000 |  |
| Zm00001d053007 | - |  |  | 1.10 | 0.029 |  |
| Zm00001d037735 | B-box zinc finger protein 22 |  |  | 1.10 | 0.001 |  |
| Zm00001d031332 | Heat shock protein 90-2 |  |  | 1.10 | 0.001 |  |
| Zm00001d043612 | Protein MARD1 |  |  | 1.10 | 0.037 |  |
| Zm00001d002565 | Zinc finger%2C C3HC4 type family protein |  |  | 1.09 | 0.005 |  |
| Zm00001d048506 | Mitochondrial import inner membrane translocase subunit TIM23-3 |  |  | 1.09 | 0.006 |  |
| Zm00001d007845 | Formin-like protein 18 |  |  | 1.09 | 0.001 |  |
| Zm00001d043735 | Bidirectional sugar transporter SWEET2 |  |  | 1.09 | 0.002 |  |
| Zm00001d017932 | Agamous-like MADS-box protein AGL16 |  |  | 1.08 | 0.006 |  |
| Zm00001d047881 | Putative DUF593 domain containing family protein |  |  | 1.08 | 0.021 |  |
| Zm00001d003277 | Stachyose synthase |  |  | 1.08 | 0.003 |  |
| Zm00001d044921 | Probable carboxylesterase 2 |  |  | 1.07 | 0.000 |  |
| Zm00001d039658 | Transcription factor HY5-like |  |  | 1.07 | 0.009 |  |
| Zm00001d047242 | Vacuolar cation/proton exchanger 2 |  |  | 1.07 | 0.037 |  |
| Zm00001d053926 | EF-hand Ca2+-binding protein CCD1 |  |  | 1.07 | 0.018 |  |
| Zm00001d024574 | Putative cytochrome P450 superfamily protein |  |  | 1.07 | 0.037 |  |
| Zm00001d028968 | Chaperone protein dnaJ 20 chloroplastic |  |  | 1.07 | 0.028 |  |
| Zm00001d051403 | plasma membrane intrinsic protein1 |  |  | 1.06 | 0.039 |  |
| Zm00001d031725 | Transcription factor TCP9 |  |  | 1.06 | 0.036 |  |
| Zm00001d009918 | Phytochrome A-associated F-box protein |  |  | 1.06 | 0.003 |  |
| novel.2161 | - |  |  | 1.05 | 0.011 |  |
| Zm00001d047685 | Probable galacturonosyltransferase-like 7 |  |  | 1.05 | 0.007 |  |
| Zm00001d028756 | - |  |  | 1.05 | 0.029 |  |
| Zm00001d032152 | cinnamoyl CoA reductase1 |  |  | 1.05 | 0.001 |  |
| Zm00001d008669 | Histone H4 |  |  | 1.04 | 0.022 |  |
| Zm00001d017817 | - |  |  | 1.04 | 0.002 |  |
| Zm00001d040783 | - |  |  | 1.04 | 0.030 |  |
| Zm00001d025964 | Putative homeobox DNA-binding and leucine zipper domain family protein |  |  | 1.04 | 0.002 |  |
| Zm00001d004095 | basic helix-loop-helix (bHLH) DNA-binding superfamily protein |  |  | 1.04 | 0.005 |  |
| Zm00001d053215 | THAUMATIN-LIKE PROTEIN 1 |  |  | 1.04 | 0.035 |  |
| Zm00001d039000 | HSP40/DnaJ peptide-binding protein |  |  | 1.04 | 0.039 |  |
| Zm00001d000310 | Protein LURP-one-related 5 |  |  | 1.03 | 0.014 |  |
| Zm00001d016570 | - |  |  | 1.03 | 0.036 |  |
| Zm00001d030678 | Transcription factor MYB48 |  |  | 1.03 | 0.003 |  |
| Zm00001d043205 | Ethylene-responsive transcription factor 4 |  |  | 1.03 | 0.038 |  |
| Zm00001d018157 | light harvesting complex a/b protein4 |  |  | 1.03 | 0.002 |  |
| Zm00001d052520 | - |  |  | 1.03 | 0.018 |  |
| Zm00001d012420 | Heat shock 70 kDa protein |  |  | 1.02 | 0.001 |  |
| Zm00001d004881 | Transcription repressor OFP6 |  |  | 1.02 | 0.025 |  |
| Zm00001d040659 | Late embryogenesis abundant protein Lea5 |  |  | 1.02 | 0.030 |  |
| Zm00001d047397 | transmembrane protein G1P-related 1 |  |  | 1.02 | 0.010 |  |
| Zm00001d047787 | Histone H2A |  |  | 1.01 | 0.037 |  |
| Zm00001d027481 | - |  |  | 1.01 | 0.002 |  |
| Zm00001d044465 | GDSL esterase/lipase |  |  | 1.00 | 0.006 |  |
| Zm00001d048422 | Photosynthetic NDH subunit of subcomplex B 2 chloroplastic |  |  | 1.00 | 0.003 |  |
| Zm00001d034715 | rubisco accumulation factor2 |  |  | 1.00 | 0.001 |  |
| Zm00001d050297 | - |  |  | -1.00 | 0.037 |  |
| Zm00001d012892 | Acyl-CoA-binding domain-containing protein 4 |  |  | -1.01 | 0.029 |  |
| Zm00001d042266 | eukaryotic initiation factor 3 gamma subunit family protein |  |  | -1.02 | 0.015 |  |
| Zm00001d035960 | DNA-directed RNA polymerase III subunit 2 |  |  | -1.04 | 0.025 |  |
| novel.5026 | PF02179:BAG domain |  |  | -1.05 | 0.009 |  |
| Zm00001d022046 | SNF2 domain-containing protein / helicase domain-containing protein / HNH endonuclease domain-containing protein |  |  | -1.06 | 0.010 |  |
| Zm00001d015692 | AUGMIN subunit 6 |  |  | -1.07 | 0.029 |  |
| Zm00001d013891 | Tubulin-folding cofactor D |  |  | -1.08 | 0.018 |  |
| Zm00001d051242 | RNA polymerase II C-terminal domain phosphatase-like 1 |  |  | -1.09 | 0.003 |  |
| Zm00001d048544 | Oxysterol-binding protein 1%3B Putative oxysterol binding domain family protein |  |  | -1.10 | 0.026 |  |
| Zm00001d045352 | Nucleosome assembly protein 1;2 |  |  | -1.11 | 0.022 |  |
| Zm00001d002250 | Probable cinnamyl alcohol dehydrogenase 1 |  |  | -1.17 | 0.032 |  |
| Zm00001d011595 | Two pore calcium channel protein 1 |  |  | -1.18 | 0.016 |  |
| Zm00001d043093 | Protein MOR1 |  |  | -1.18 | 0.019 |  |
| Zm00001d036741 | Isoleucine--tRNA ligase cytoplasmic |  |  | -1.19 | 0.010 |  |
| Zm00001d037772 | catalytics |  |  | -1.22 | 0.037 |  |
| Zm00001d052316 | Ferritin-1%2C chloroplastic |  |  | -1.23 | 0.002 |  |
| Zm00001d002679 | Mechanosensitive ion channel protein 6 |  |  | -1.24 | 0.034 |  |
| Zm00001d027472 | Callose synthase 3 |  |  | -1.24 | 0.001 |  |
| Zm00001d049379 | tetratricopeptide repeat (TPR)-containing protein |  |  | -1.24 | 0.023 |  |
| Zm00001d025793 | Gamma-tubulin complex component 2 |  |  | -1.27 | 0.018 |  |
| Zm00001d033993 | DExH-box ATP-dependent RNA helicase DExH16 mitochondrial |  |  | -1.27 | 0.047 |  |
| Zm00001d039563 | ARM repeat superfamily protein |  |  | -1.28 | 0.003 |  |
| Zm00001d019108 | Ribosomal protein S1-like1 |  |  | -1.33 | 0.003 |  |
| Zm00001d038917 | ARM repeat superfamily protein |  |  | -1.33 | 0.038 |  |
| Zm00001d007117 | GTP-binding protein OBGC chloroplastic |  |  | -1.37 | 0.002 |  |
| Zm00001d040599 | Quinolinate synthase chloroplastic |  |  | -1.37 | 0.014 |  |
| Zm00001d016037 | Adenine nucleotide alpha hydrolases-like superfamily protein |  |  | -1.44 | 0.014 |  |
| Zm00001d050874 | NAP1-related protein 2 |  |  | -1.44 | 0.020 |  |
| novel.4562 | - |  |  | -1.49 | 0.008 |  |
| Zm00001d039301 | Vegetative storage protein PNI288 |  |  | -1.51 | 0.037 |  |
| Zm00001d002475 | Binding partner of ACD11 1 |  |  | -1.56 | 0.029 |  |
| Zm00001d005937 | - |  |  | -1.57 | 0.025 |  |
| Zm00001d033180 | brassinosteroid-deficient dwarf1 |  |  | -1.58 | 0.025 |  |
| novel.4603 | - |  |  | -1.68 | 0.002 |  |
| novel.2307 | PF03398:Regulator of Vps4 activity in the MVB pathway |  |  | -1.69 | 0.011 |  |
| Zm00001d038475 | ribosome export associated1 |  |  | -1.71 | 0.014 |  |
| Zm00001d053735 | Protein kinase superfamily protein |  |  | -1.72 | 0.012 |  |
| Zm00001d048207 | Putative RING zinc finger domain superfamily protein |  |  | -1.72 | 0.008 |  |
| Zm00001d016175 | Homeodomain-like superfamily protein |  |  | -1.73 | 0.046 |  |
| Zm00001d047453 | Villin-2 |  |  | -1.88 | 0.048 |  |
| Zm00001d036062 | Exocyst complex component SEC5A |  |  | -1.94 | 0.008 |  |
| Zm00001d000002 | dull endosperm1 |  |  | -2.04 | 0.044 |  |
| Zm00001d042027 | - |  |  | -2.06 | 0.048 |  |
| Zm00001d029410 | Serine carboxypeptidase-like 51 |  |  | -2.13 | 0.012 |  |
| Zm00001d023779 | 3-hydroxyisobutyryl-CoA hydrolase 1 |  |  | -2.20 | 0.042 |  |
| Zm00001d034713 | Anthranilate synthase alpha subunit 2 chloroplastic |  |  | -2.24 | 0.000 |  |
| Zm00001d018617 | gibberellin 2-oxidase12 |  |  | -2.37 | 0.026 |  |
| Zm00001d037951 | Serine/threonine-protein kinase TOR |  |  | -2.47 | 0.023 |  |
| Zm00001d000294 | O-succinylhomoserine sulfhydrylase |  |  | -2.56 | 0.016 |  |
| Zm00001d037005 | Putative FAD-binding Berberine family protein |  |  | -2.72 | 0.046 |  |
| Zm00001d008173 | Peroxidase 2 |  |  | -2.82 | 0.000 |  |
| Zm00001d036014 | E3 ubiquitin-protein ligase XBAT33 |  |  | -2.88 | 0.010 |  |
| Zm00001d047930 | - |  |  | -3.05 | 0.002 |  |
| Zm00001d043512 | Protein kinase family protein with leucine-rich repeat domain |  |  | -3.31 | 0.041 |  |
| Zm00001d012575 | Protein MICRORCHIDIA 6 |  |  | -3.59 | 0.003 |  |
| Zm00001d021695 | O-Glycosyl hydrolases family 17 protein |  |  | -3.84 | 0.042 |  |
| Zm00001d037607 | Probable WRKY transcription factor 50 |  |  | -3.89 | 0.037 |  |
| Zm00001d054096 | L-ascorbate peroxidase 2 cytosolic |  |  | -3.98 | 0.027 |  |
| Zm00001d019399 | Putative lipid-transfer protein DIR1 |  |  | -4.01 | 0.041 |  |
| Zm00001d040126 | - |  |  | -4.72 | 0.018 |  |
| Zm00001d017991 | - |  |  | -6.26 | 0.005 |  |
| novel.5260 | PF10536:Plant mobile domain |  |  | -7.03 | 0.008 |  |
| Zm00001d019400 | PVR3-like protein |  |  | -7.20 | 0.007 |  |
| Zm00001d017669 | Peptide chain release factor PrfB1 chloroplastic |  |  | -7.74 | 0.000 |  |
| Foliar spraying with ABA started on the first day when the pot mix was at the upper threshold of 50% for moderate drought. The results were based on transcriptome sequencing of the second fully-expanded leaves down from the top of 3 individual plants 9 d after the pot mix moisture was at the upper threshold of 50% for moderate drought. ^a^ Positive and negative values indicated up-regulation and down-regulation of gene expression, respectively. ABA, Abscisic acid; DEG, Differentially expressed gene; padj: Adjust *p*-value; Y478, Maize inbred line Ye478. | | | | | | |
