# Supplementary material for: Characterization of ZmPMP3g function in drought tolerance of maize
Source: Sci Rep. 2023 May 5;13:7375. doi: 10.1038/s41598-023-32989-4 (PMC10163268; doi:10.1038/s41598-023-32989-4)
Supplement: Supplementary file 7 — Supplementary Table S3. [file 41598_2023_32989_MOESM7_ESM.docx]

| **Table S3** The DEGs in leaves of non-transgenic wild type Y478 under a combination of control and exogenous ABA as a combination of control and exogenous ABA (Y478) vs. control (Y478) in pot experiments with foliar spraying with exogenous ABA | | | | | | |
| --- | --- | --- | --- | --- | --- | --- |
| Gene ID |  | Gene description |  | log2 (fold change)^a^ | padj |  |
| Zm00001d042779 |  | Protein ABSCISIC ACID-INSENSITIVE 5 |  | 2.18 | 0.003 |  |
| Zm00001d014029 |  | myb domain protein 15 |  | 1.99 | 0.041 |  |
| Zm00001d002143 |  | bZIP-transcription factor 27 |  | 1.26 | 0.026 |  |
| Zm00001d025544 |  | Zeaxanthin epoxidase chloroplastic |  | -1.23 | 0.000 |  |
| Foliar spraying with ABA started on the first day when the pot mix was at the upper threshold of 50% for moderate drought. The results were based on transcriptome sequencing of the second fully-expanded leaves down from the top of 3 individual plants 9 d after the pot mix moisture was at the upper threshold of 50% for moderate drought. ^a^ Positive and negative values indicated up-regulation and down-regulation of gene expression, respectively. ABA, Abscisic acid; DEG, Differentially expressed gene; padj: Adjust *p*-value; Y478, Maize inbred line Ye478. | | | | | | |
